# First-Principles Study of Magnesium-Induced Phase Stabilization in Gallium Oxide Polymorphs

**DOI:** 10.1021/acs.chemmater.6c01704

**Published:** 2026-08-04

**Authors:** Viswesh Prakash, Jingyu Tang, Lisa M. Porter, Rachel C. Kurchin

**Affiliations:** Department of Materials Science and Engineering, 2167 Carnegie Mellon University, Pittsburgh, Pennsylvania 15213, United States

## Abstract

In this study, we investigate the effect of Mg incorporation on the relative phase stability of the four primary Ga_2_O_3_ polymorphs using density functional theory (DFT) calculations, with the goal of rationalizing experimental observations suggesting that diffusion from MgAl_2_O_4_ substrates contributes to the relative stabilization of the γ phase. Mg incorporation is modeled up to 25% of Ga sites within supercells derived from fully relaxed unit cells of each polymorph. Our results show that, while β-Ga_2_O_3_ remains the thermodynamically most stable phase, the enthalpy and free energy differences between polymorphs decrease with increasing Mg content. The inherently disordered γ phase, with its high configurational entropy, becomes less energetically unfavorable under Mg substitution, suggesting that entropy-driven stabilization may facilitate its formation under high-temperature and/or nonequilibrium growth conditions, such as those previously reported. These findings provide a thermodynamic rationale for the experimental observation of the γ phase during epitaxial growth on MgAl_2_O_4_ spinel substrates.

## Graphical abstract

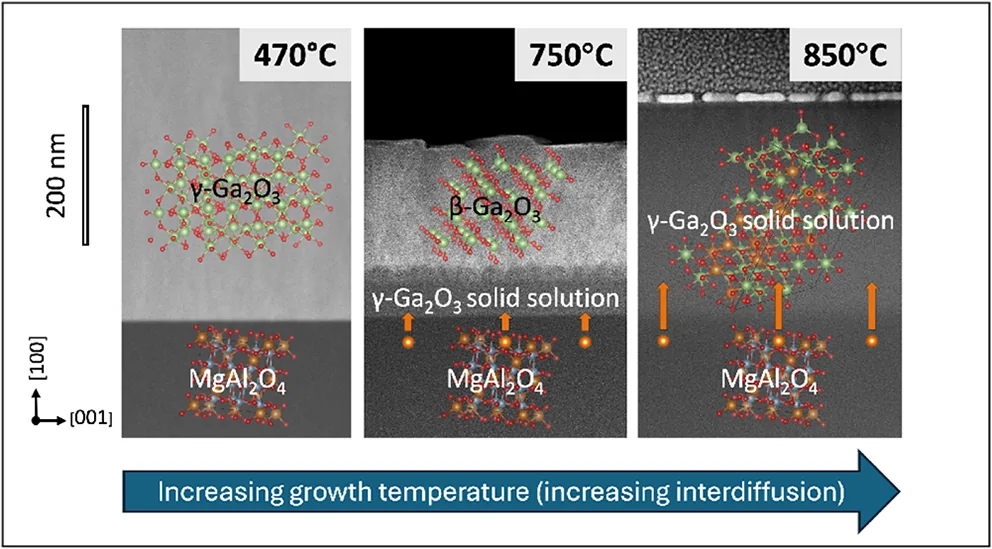

## Introduction

Gallium oxide (Ga_2_O_3_) has emerged as a material of considerable interest for high-power electronic applications, owing to its ultrawide bandgap, high breakdown field, and potential for efficient power device performance.^1–4^ Ga_2_O_3_ has four widely accepted polymorphs showing the following symmetries: α (trigonal), β (monoclinic), γ (cubic), and κ (also referred to as ϵ, orthorhombic). Among these, β-Ga_2_O_3_ is the thermodynamically stable phase,^5^ while γ-Ga_2_O_3_ is consistently identified as the least stable based on density functional theory (DFT) calculations.^6,7^

Despite its theoretical instability, the γ phase is frequently observed in experimental studies. Notably, γ-Ga_2_O_3_ has been reported to form as inclusions during the epitaxial growth of β-Ga_2_O_3_ films on β-Ga_2_O_3_ substrates, particularly when alloyed with Al_2_O_3_ or following ion implantation.^8,9^ Additionally, thin γ-Ga_2_O_3_ layers—typically on the order of a few tens of nanometers—have been detected at the interfaces of β-Ga_2_O_3_ films grown on MgO substrates with various crystallographic orientations, including (001), (011), and (111).^10,11^ These observations suggest that specific growth conditions and substrate interactions can kinetically favor the formation of the metastable γ phase.

Experimental evidence also indicates that the incorporation of certain elements during film growth can contribute to the stabilization of the γ phase. In particular, the addition of Mg,^12–14^ Al,^15^ Mn,^16–18^ Fe,^19^ or Cu^20^ has been found to promote the formation or retention of the γ phase. Our previous studies revealed the sequence of γ-Ga_2_O_3_ → β-Ga_2_O_3_ → γ-Ga_2_O_3_ solid solution on (100)-oriented MgAl_2_O_4_ substrates grown by metal–organic chemical vapor deposition (MOCVD) as growth temperature is increased. Specifically, γ-Ga_2_O_3_ formed at 470 °C, followed by the emergence of β-Ga_2_O_3_ in the temperature range of 530–650 °C. At higher growth or annealing temperatures (>750 °C), substantial Mg and Al diffusion occurred, leading to the formation of a γ-Ga_2_O_3_ solid-solution.^14,21,22^ Scanning transmission electron microscopy (STEM) analyses conducted across the substrate/thin-film interfaces of these films are shown in Figure 1 (data were collected as part of the same studies reported in these prior works). In this figure, it can be observed that at the intermediate growth temperature (750 °C), β-Ga_2_O_3_ becomes the preferred phase in the upper portion of the film where Mg and Al incorporation is negligible, while a transition layer below with substantial Mg and Al incorporation exhibits the γ structure. However, by 850 °C, diffusion is fast enough that this β layer is no longer present, and γ-phase solid solution is stabilized throughout the film. More detailed growth conditions and structural characterizations of these films can be found in our previous publications.^14,21,22^

**Figure 1:**
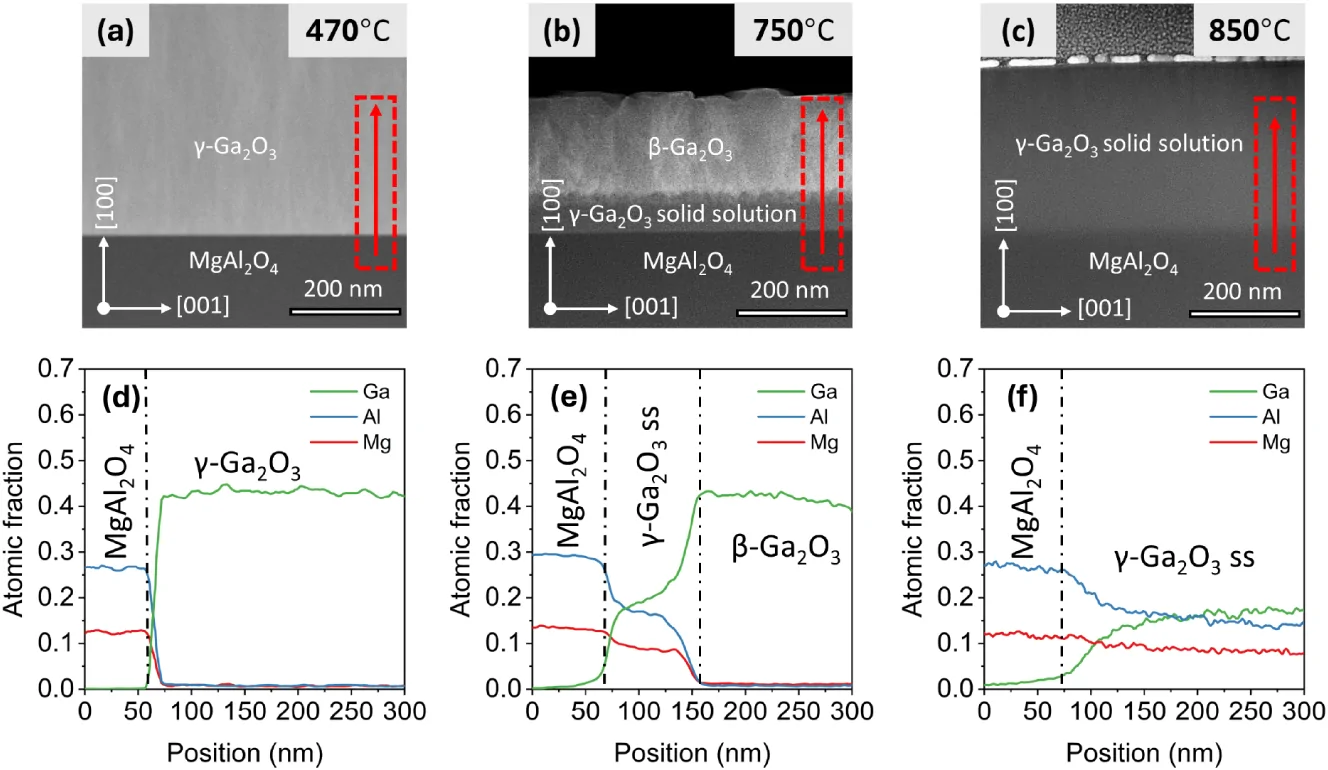
Cross-sectional high-angle annular dark-field scanning transmission electron microscopy (HAADF-STEM) images of films grown at (a) 470 °C, (b) 750 °C, and (c) 850 °C, respectively and their corresponding integrated energy dispersive X-ray spectrometry (EDX) composition profiles shown in (d–f). The red dashed rectangles with arrows indicate the regions and directions along which the EDX profiles were acquired. “γ-Ga_2_O_3_ ss” refers to a solid solution.

Further evidence for the role of MgAl_2_O_4_ substrates in stabilizing the γ phase has been provided by Oshima et al.^23^ That study reports X-ray diffraction peak shifts indicative of diffusion-stabilized γ-Ga_2_O_3_ formation in films grown via mist chemical vapor deposition (mist-CVD) on (100)-oriented MgAl_2_O_4_. Hou et al.^12,13^ also reported the successful growth of (111)-oriented, phase-pure Mg_*x*_Ga_2_O_4_ inverse spinel films on (0001) sapphire substrates, without the formation of the β phase. Furthermore, they observed that postgrowth annealing at 900 °C led to improved structural ordering without inducing a phase transformation to the β structure.

In addition to experimental demonstrations,^15,24^ theoretical analyses indicate that the γ phase can become the dominant structural form in (Al_*x*_Ga_1–*x*_)_2_O_3_ films depending on the Al concentration and specific growth technique employed. Several computational studies have sought to elucidate the effects of alloying on the phase stability of Ga_2_O_3_ polymorphs. Peelaers et al.^25^ investigated the relative stability of monoclinic (β) and corundum (α) (Al_*x*_Ga_1–*x*_)_2_O_3_ alloys at zero temperature, while Seacat et al.^26^ extended this analysis to the orthorhombic (κ) phase. Mu and Van de Walle^27^ conducted a comprehensive analysis of Al incorporation into all known Ga_2_O_3_ polymorphs, demonstrating that alloying with Al significantly reduces the energy difference between the γ and β phases, thereby enhancing the metastability of γ-Ga_2_O_3_.

While the stabilizing effect of Al alloying on the γ phase has been explored in detail, experimental observations suggest that both Al and Mg play critical roles in stabilizing the γ phase under specific growth conditions. However, the independent influence of Mg incorporation on the phase stability of Ga_2_O_3_ polymorphs remains an open question and is largely unaddressed in the existing literature.

The present study seeks to address this gap by employing a first-principles computational approach based on DFT. We investigate the relative phase stability of all four Ga_2_O_3_ polymorphs—α, β, γ, and κ—in (Mg_*x*_Ga_1–*x*_)_2_O_3–*x*_ alloys at zero temperature. Through this work, we aim to clarify the thermodynamic role of Mg incorporation in stabilizing the metastable γ phase and to contribute a more complete understanding of the compositional factors that govern polymorph formation in Ga_2_O_3_-based materials. Future work may examine interactions between Al and Mg that would likely become important at higher alloying concentrations where the assumption of independence could break down.

## Methodology

### Structures of Polymorphs

In this study, we focus on the four commonly accepted polymorphs of Ga_2_O_3_—β (monoclinic), κ (orthorhombic), α (trigonal), and γ (cubic)—as well as the MgO structure, which adopts a cubic rock-salt configuration. Among these Ga_2_O_3_ polymorphs, the γ phase is characterized by a disordered structure,^7,28^ where cation vacancies can occupy both tetrahedral and octahedral sites in the defective spinel structure. Hence the unit cell of γ-Ga_2_O_3_ was constructed following the two-step process outlined in the literature.^6,27,29^ First, a triclinic supercell was derived from the conventional cubic spinel cell corresponding to the stoichiometry Ga_3_O_4_ by applying a lattice transformation to the lattice vectors (*a⃗*_0_, *b⃗*_0_, *c⃗*_0_) as follows:

$$\left(\begin{array}{l} {\begin{array}{c} \mathrm{a⃗} \end{array}}^{\prime }\\ {\begin{array}{c} \mathrm{b⃗} \end{array}}^{\prime }\\ {\begin{array}{c} \mathrm{c⃗} \end{array}}^{\prime } \end{array}\right)=\left(\begin{array}{lll} 0.5 & 0.5 & 0\\ 0 & 0.5 & 0.5\\ 1.5 & 0 & 1.5 \end{array}\right)\left(\begin{array}{l} {\mathrm{a⃗}}_{0}\\ {\mathrm{b⃗}}_{0}\\ {\mathrm{c⃗}}_{0} \end{array}\right)$$ 1

Subsequently, two Ga atoms were selectively removed from octahedral sites in the transformed triclinic cell, thereby yielding the desired stoichiometry of Ga_2_O_3_. These Ga atoms were chosen such that they are separated by a distance of approximately 7.9 Å. The unit cells for all the Ga_2_O_3_ polymorphs are illustrated in Figure 2. The types of cation and anion sites characteristic of each polymorph are summarized in Table 1.

**Figure 2:**
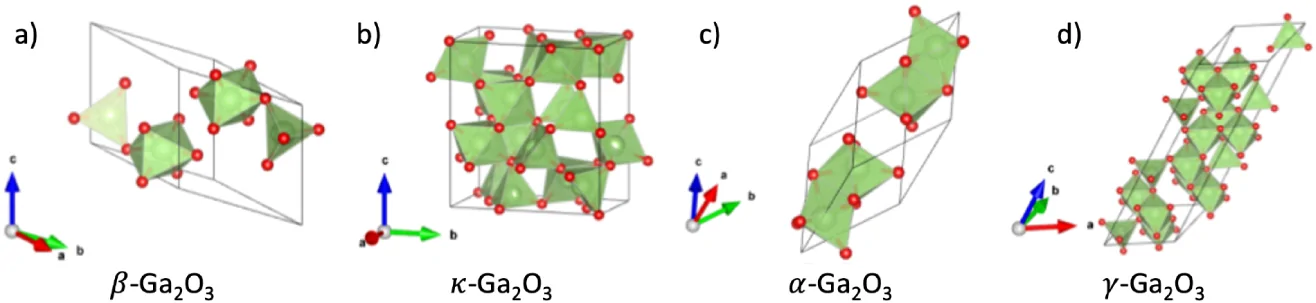
Illustration of structures for Ga_2_O_3_ polymorphs in decreasing order of stability as determined by DFT—(a) β-Ga_2_O_3_; (b) κ-Ga_2_O_3_; (c) α-Ga_2_O_3_; (d) γ-Ga_2_O_3_. Red spheres denote O atoms and green spheres and polyhedra denote Ga atoms and their coordination. Cells (a) and (c) are primitive unit cells of the respective structures. Structural visualization was performed using VESTA.^30^

**Table 1:** Summary of Ga Coordination and O Site Connectivity for Structures of All Ga_2_O_3_ Polymorphs

Structure	Ga site ratio (octahedral:tetrahedral)	Types of O sites [O at corners of polyhedra]
β	1:1	Edge sharing between octahedra
		Corner sharing between tetrahedra
		Corner sharing between octahedra and tetrahedra
κ	3:1	Edge sharing between octahedra
		Corner sharing between tetrahedra
		Corner sharing between octahedra and tetrahedra
α	All octahedral	Edge sharing between octahedra
γ	5:3	Edge sharing between octahedra
		Corner sharing between tetrahedra
		Corner sharing between octahedra and tetrahedra

To examine the effects of Mg incorporation, we construct alloyed structures by substituting Ga atoms with Mg within supercells derived from the respective unit cells shown in Figure 2. At each specified Mg concentration, various configurations can be generated by selectively replacing Ga atoms occupying distinct cation sites. To preserve charge balance, oxygen vacancies are introduced alongside Mg substitution (see Computational Details for more information). While there are in principle other mechanisms for charge compensation, prior studies have established that the introduction of deep acceptors such as Mg (and consequent shifting of the Fermi level toward the valence band maximum) strongly favors the formation of $V_{O}^{2+}$ .^31,32^ While that work focused on β-Ga_2_O_3_, the similar local coordination environments and associated electronic structure make this a reasonable assumption for other polymorphs as well. We also note that prior work^33^ has established that especially in disordered Ga_2_O_3_ networks (the γ phase is partially disordered), oxygen is remarkably mobile, even at relatively modest temperatures such as 300–370 °C. Thus, oxygen escaping the film following vacancy formation is unlikely to be difficult.

In this study, we restrict the extent of Mg substitution to a maximum of 25% of the available Ga sites.

### Computational Details

All first-principles calculations were performed using DFT within the projector augmented-wave^34^ (PAW) formalism as implemented in the Quantum ESPRESSO package. The exchange-correlation interactions were described using the generalized gradient approximation (GGA) as formulated by Perdew, Burke, and Ernzerhof (PBE).^35^ Ion-electron interactions were described using the Standard Solid-State Pseudopotentials (SSSP) library.^36^ Prior to structural relaxations, convergence tests were carried out to determine appropriate values for the plane-wave kinetic energy cutoff, the charge density kinetic energy cutoff, and the Brillouin-zone sampling mesh. Based on these tests, the plane-wave energy cutoff was fixed at 140 Ry, and the charge density cutoff was set to 560 Ry. Brillouin-zone integration was performed using a Γ-centered Monkhorst–Pack 6 × 6 × 6 k-point mesh for the primitive cells of the monoclinic β and trigonal α phases, a 6 × 4 × 4 mesh for the orthorhombic κ phase, and a 6 × 6 × 2 mesh for the triclinic cell of the defective spinel γ phase. All polymorphs were fully relaxed with respect to both atomic positions and lattice parameters. The resulting optimized structural parameters are summarized in Table 2 and are in good agreement with experimentally reported values.

**Table 2:** DFT-Relaxed Structural Parameters for All Polymorphs of Ga_2_O_3_ in Comparison to Experimentally Determined Lattice Parameters^a^

	Experimental	Calculated
Mononclinic (β)^37^		
a	12.2	12.4565
b	3.0	3.085
c	5.8	5.879
beta	104	103.688
Trigonal (α)^38^		
a	4.98	5.0597
c	13.43	13.6283
Orthorhombic (κ)^39^		
a	5.0	5.1243
b	8.68	8.8023
c	9.23	9.4124
Defective spinel (γ)^28^		
a	8.24	8.325

a Lattice parameters in Å and angles in degrees.

To minimize the effects of short-range ordering induced by periodic boundary conditions in DFT calculations of substitutional alloys, supercells were constructed for all polymorphs based on the respective unit cells shown in Figure 2. Furthermore, to avoid ordering along specific lattice directions, supercells were constructed using lattice transformation matrices that yield approximately cubic supercells with a comparable distribution of Ga sites along each axis of the supercell. These lattice transformations are carried out as follows:

$$\left(\begin{array}{l} {\begin{array}{c} \mathrm{a⃗} \end{array}}^{\prime }\\ {\begin{array}{c} \mathrm{b⃗} \end{array}}^{\prime }\\ {\begin{array}{c} \mathrm{c⃗} \end{array}}^{\prime } \end{array}\right)=A_{\mathrm{sc}}\left(\begin{array}{l} \mathrm{a⃗}\\ \mathrm{b⃗}\\ \mathrm{c⃗} \end{array}\right),$$ 2

where *a⃗*, *b⃗*, and *c⃗* are the lattice vectors of the primitive cell, *A*_sc_ is the integer lattice transformation matrix, and ${\begin{array}{c} \mathrm{a⃗} \end{array}}^{\prime }$ , ${\begin{array}{c} \mathrm{b⃗} \end{array}}^{\prime }$ , and ${\begin{array}{c} \mathrm{c⃗} \end{array}}^{\prime }$ are the resulting lattice vectors of the generated supercell, expressed as linear combinations of the original lattice vectors.

The lattice transformation matrices used for the β, α, and κ phases are as follows:

$$A_{\beta }=\left(\begin{array}{lll} 2 & 0 & 0\\ -4 & 4 & 0\\ 0 & 0 & 2 \end{array}\right)$$

$$A_{\alpha }=\left(\begin{array}{lll} 2 & 0 & 0\\ -1 & 3 & -1\\ -1 & -1 & 3 \end{array}\right)$$

$$A_{\kappa }=\left(\begin{array}{lll} 2 & 0 & 0\\ 0 & 1 & 0\\ 0 & 0 & 1 \end{array}\right)$$

For the defective spinel γ-Ga_2_O_3_ having a primitive cell showing triclinic symmetry, we adopt the method of constructing an oriented bulk structure as described by Sun and Ceder,^40^ to ensure that the out-of-plane lattice vector aligns with experimentally observed orientations. The resulting transformation matrix from this approach is

$$A_{\gamma }=\left(\begin{array}{lll} 0 & 2 & 0\\ -3 & 0 & 1\\ 2 & -1 & 0 \end{array}\right)$$

Applying the aforementioned lattice transformations to the unit cells results in supercells containing 160 atoms for the β, α, and γ polymorphs, and 80 atoms for the κ polymorph. These supercells serve as the basis for modeling Mg incorporation into the Ga_2_O_3_ lattice by substituting Ga atoms with Mg. Since Mg^2+^ has a lower valence state than Ga^3+^, charge compensation is required to maintain overall neutrality. This is achieved by introducing one oxygen vacancy for every two Ga sites substituted with Mg. Thus, the alloyed compositions studied in this work follow the general formula (Mg_*x*_Ga_1–*x*_)_2_O_3–*x*_.

Mg substitution was explored both with and without cation site preference, i.e., Mg atoms are substituted at Ga sites both at random and by restricting to Ga atoms occupying a specific cation site (octahedral or tetrahedral). To mitigate a combinatorial explosion of possible configurations to calculate, we downsample to a representative subset. To select this subset of configurations for a given polymorph and Mg concentration, we adopt a statistical approach based on radial distribution functions (RDFs). Initially, a large number of inequivalent structures are generated. By computing all partial RDF’s between species (Ga, Mg, O, and vacancies) within the structure and comparing results from subsets to averages over the full ensemble, we can select a subset that retains acceptable representation of the full range of environments. For more details, see the Supporting Information.

The thermodynamic stability of the Mg-incorporated structures at zero temperature is evaluated through the calculation of their enthalpies of formation (ΔH). This is done by referencing the total energies of the substituted structures to the most stable phases of the constituent binary oxides—namely, β-Ga_2_O_3_ and MgO. The formation enthalpy for a given composition of the alloyed system (Mg_*x*_Ga_1–*x*_)_2_O_3–*x*_ is computed according to the following expression (where *E*[···] refers to DFT total energy):

3 $$\Delta H\left[{\left({\mathrm{Mg}}_{x}{\mathrm{Ga}}_{1-x}\right)}_{2}O_{3-x}\right]=E\left[{\left({\mathrm{Mg}}_{x}{\mathrm{Ga}}_{1-x}\right)}_{2}O_{3-x}\right]-\left(1-x\right)E\left[{\mathrm{Ga}}_{2}O_{3}\right]-2xE\left[\mathrm{MgO}\right]$$

Gibbs free energies (Δ*G* = Δ*H* – *T*Δ*S*) were computed using the enthalpy above and configurational entropies (while other sources of entropy may be non-negligible, we expect their differences between phases to be modest), which were computed relative to pure Ga_2_O_3_ according to

4 $$\Delta S=-k_{B}\underset{i}{\sum }N_{i}\underset{j}{\sum }x_{j}\ln x_{j},$$

where *x* is a concentration per formula unit, *i* indexes sublattice types (cations or anions), *N*_*i*_ is the number of sites on that sublattice per formula unit, and *j* indexes species that can occupy that sublattice: Ga and Mg (and vacancies for the γ phase) on cation sites, and O and vacancies on anion sites.

## Results and Discussion

The phase stability of the different Ga_2_O_3_ polymorphs was assessed by comparing their enthalpies of formation (Δ*H*). Our results for the unsubstituted Ga_2_O_3_ structures agree with previous calculations^6^ and the relative phase stability follows the expected trend, in order of increasing Δ*H*: β < κ < α < γ. The γ phase, as anticipated, is the least stable, with a Δ*H* of 0.222 eV/f.u.—significantly higher than those of the α phase (Δ*H* = 0.153 eV/f.u.) and κ phase (Δ*H* = 0.106 eV/f.u.), all referenced to the β phase. Our calculated Δ*H* values are consistent with prior literature,^25–27^ deviating by ∼0.01 eV/f.u. Figure 3 presents the minimum Δ*H* values for the β, κ, and α polymorphs as a function of Mg substitution on Ga sites, without cell shape relaxation. From Figure 3, we note that as the Mg concentration increases, the differences in enthalpy of formation across the polymorphs decrease with increasing concentration of Mg. This suggests that Mg incorporation reduces the energetic penalty for forming the metastable polymorphs, rendering them more competitive in energy with the thermodynamically stable β phase. Consequently, the addition of Mg may make the metastable polymorphs energetically accessible structures that can be grown by tuning the growth conditions, where the influence of kinetics, strain or codoping effects of other atoms could further stabilize the different polymorphs.

**Figure 3:**
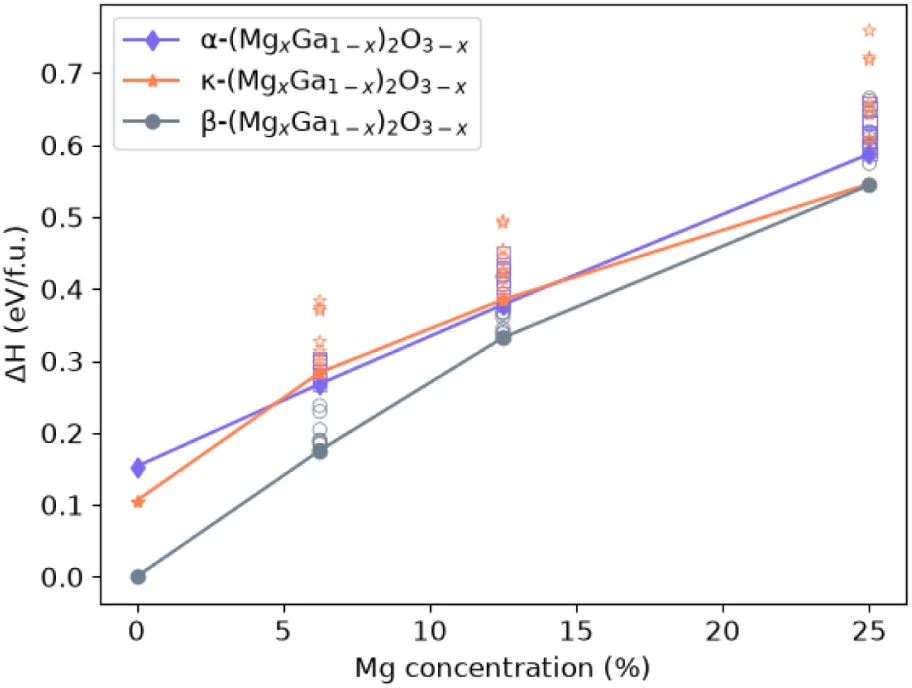
Bulk enthalpy of formation (Δ*H*) for (Mg*_x_* Ga_1–*x*_)_2_O_3–*x*_ alloys as a function of Mg concentration (% of Ga sites substituted) for the α-, β-, and κ-polymorphs of Ga_2_O_3_. Filled symbols represent minimum values, while open symbols represent the values for enthalpy of formation of other structures in the selected sample of structures for each polymorph at a given concentration.

Given that both the γ and β phases have been experimentally observed to grow on Mg-containing substrates such as MgO and MgAl_2_O_4_, we examine the effect of Mg substitution in these two polymorphs in greater detail. Specifically, we analyze the site preference and energetic impact of Mg incorporation across different substitution scenarios. To ensure relevance to experimentally observed growth conditions, these calculations are performed using epitaxially constrained structures, where the out-of-plane lattice vector that was allowed to relax is consistent with the experimentally reported out-of-plane normal for epitaxial growth (and the other two lattice vectors were fixed according to the epitaxial matching constraint). Our results indicate that in β-Ga_2_O_3_, Mg atoms show a clear preference to occupy octahedral Ga sites. Figure 4a shows the average and range of the enthalpy of formation for substituted β-Ga_2_O_3_ as a function of the Mg concentration. We see that the octahedral substitution of Mg significantly reduces the enthalpy of formation for the substituted structure at higher concentrations when compared to tetrahedral substitution or random substitution. In contrast, γ-Ga_2_O_3_ does not exhibit a strong site preference for Mg; the enthalpy of formation remains relatively insensitive to the specific site occupancy across the full range of Mg concentrations considered. This can be seen in Figure 4b, exhibiting no noticeable reduction in the enthalpy of formation for any given site occupancy of Mg in the γ structure.

**Figure 4:**
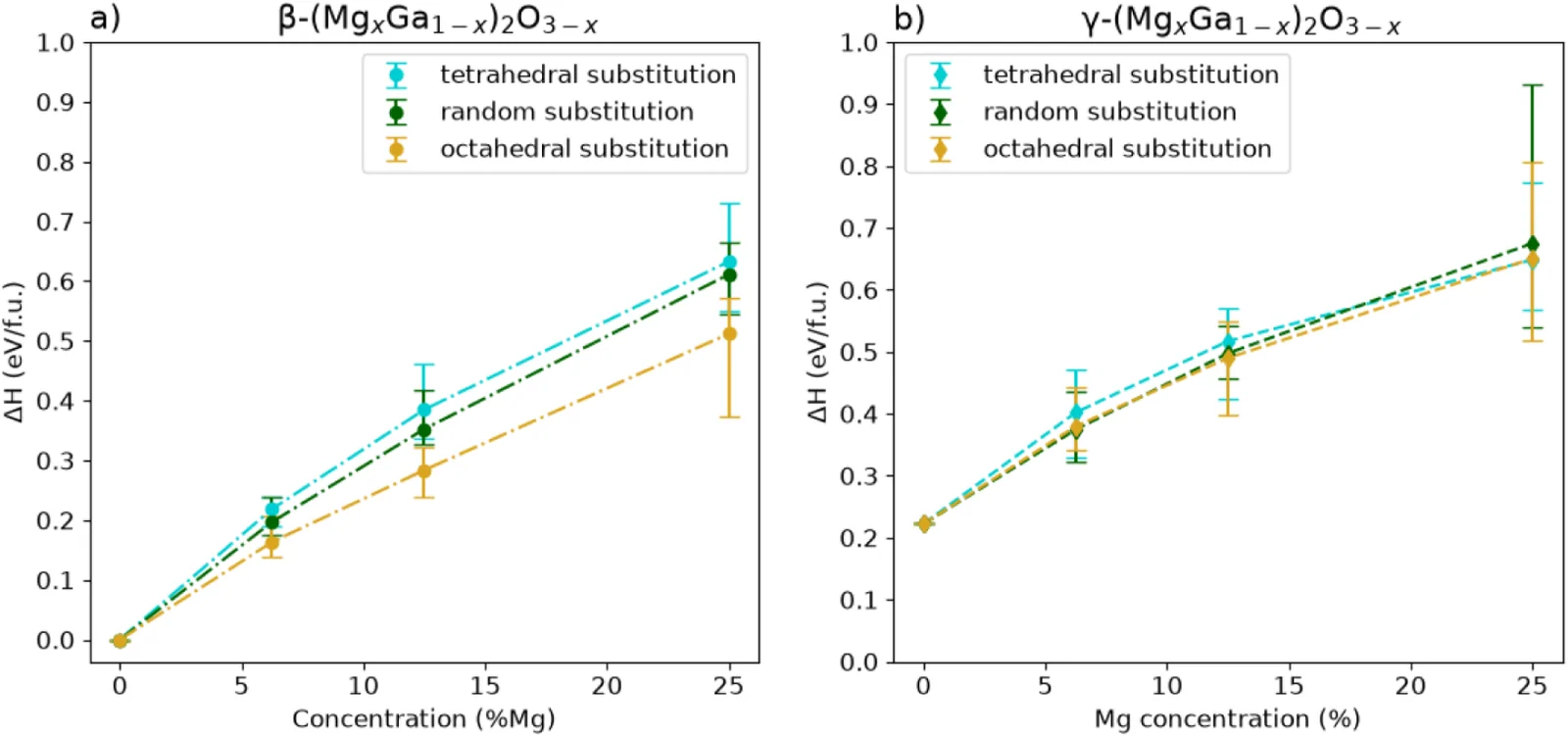
Average and range of enthalpy of formation (Δ*H*) for epitaxially strained Mg-substituted (a) β-Ga_2_O_3_ and (b) γ-Ga_2_O_3_ as a function of % Ga sites substituted, shown for three site occupancy configurations: octahedral-only (yellow), tetrahedral-only (blue), and random distribution (green).

To further understand the energetic relationship between the γ and β phases during Mg substitution, we directly compare the enthalpy of formation for both polymorphs as a function of Mg concentration for the same three substitution configurations: octahedral-only, tetrahedral-only, and random occupancy (Figure S20). The key takeaway from this comparison is that, like in the relaxed bulk results shown in Figure 3, Mg incorporation also reduces the energy difference between the β and γ phases in these strained simulations.

Figures 5a–c show atomic-scale HAADF-STEM images of representative regions from the same three films shown in Figure 1, all exhibiting structural features consistent with the spinel structure shown in Figure 5d. Three line profiles highlighted in orange, red and blue, each starting and ending at atoms on octahedral sites, were extracted from the images to evaluate the cation site preference based on the contrast difference between tetrahedral and octahedral sites, where the HAADF contrast is roughly *Z* ^1.6^ (where *Z* is the atomic number). The lighter Al and Mg atoms preferentially occupy octahedral Ga sites and the heavier Ga atoms prefer to be on tetrahedral sites, as observed by the inversion of the image intensity profiles (Figure 5e) for pure γ-Ga_2_O_3_ (Figure 5a, orange line in e) and γ-Ga_2_O_3_ solid solutions (Figures 5b–c, red/blue lines in e).

**Figure 5:**
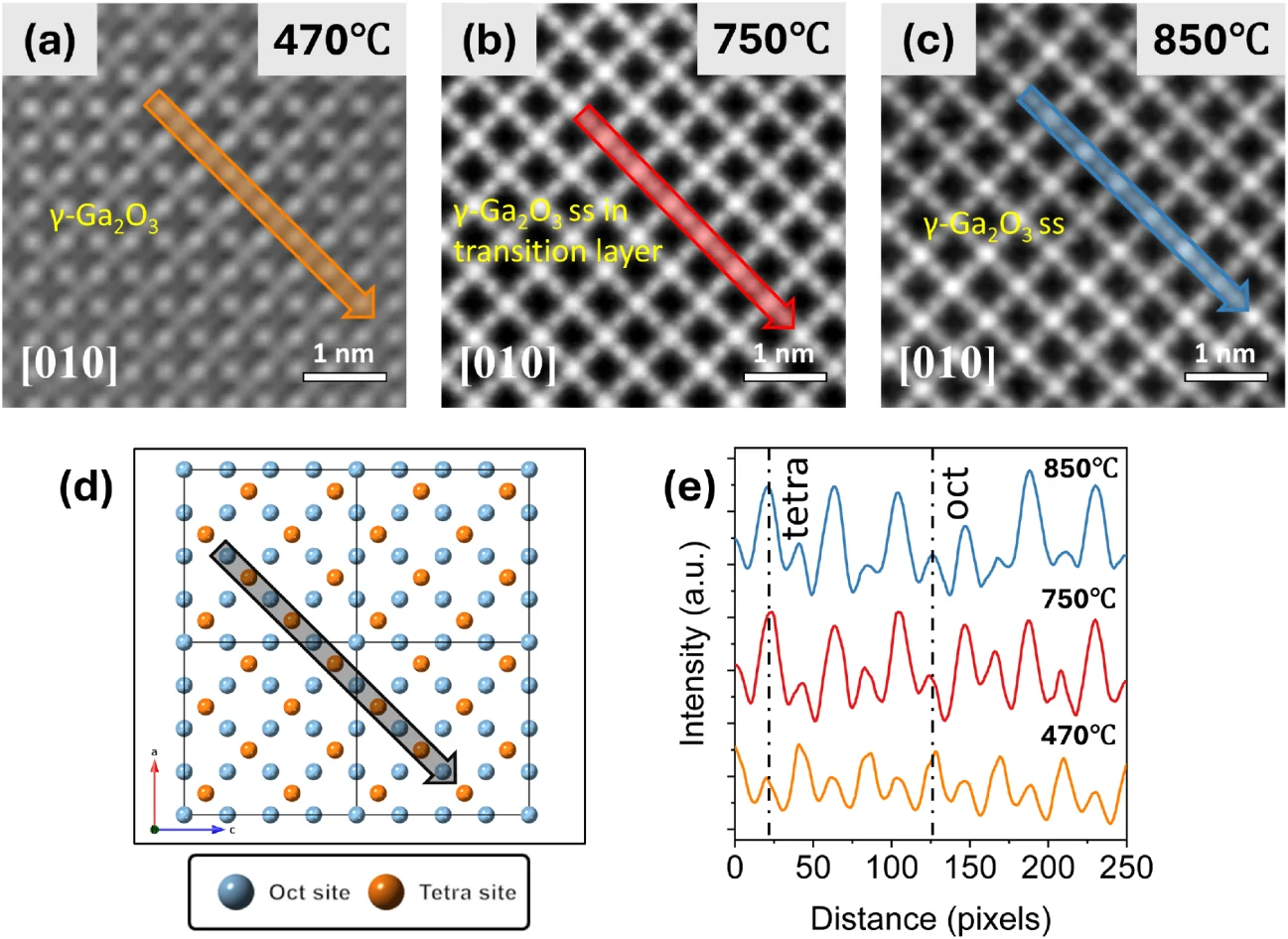
Atomic-scale HAADF-STEM images for the film grown at (a) 470 °C, (b) 750 °C and (c) 850 °C, respectively. Subfigure (d) shows the atomic structure of cubic spinel structure along [010] zone, while (e) shows the combined line profiles of the image intensity across three atomic rows from (a) to (c), showing the inversion in relative contrast between tetrahedral and octahedral sites.

In a study similar to the present work, Mu et al.^27^ report that Al strongly prefers to occupy octahedral Ga sites in both β- and γ-Ga_2_O_3_, unlike the results reported here for Mg, where this preference is only present in the β phase. While Mg shows no strong site preference in γ-Ga_2_O_3_, its incorporation does reduce the enthalpy difference between the β and γ phases, and the diffusion of Al and Mg during growth collectively make the γ phase more energetically accessible. This is consistent with experimental observations that show Al preferentially occupies octahedral sites while Ga tends to occupy tetrahedral sites. Given that the γ phase contains a higher proportion of octahedral to tetrahedral sites (5:3), and Mg does not show a site preference, it is more likely to occupy an octahedral site while stabilizing the γ phase.

So far, we have only discussed enthalpies of formation—it is important to consider that free energy, rather than enthalpy alone, governs phase stability under real synthesis conditions. In particular, configurational entropy, which arises from the number of ways atoms can be arranged in a structure, can play a significant role in stabilizing disordered or metastable phases at finite temperatures. The γ-Ga_2_O_3_ phase is inherently structurally disordered, characterized by a defective spinel lattice with partial occupancy of both tetrahedral and octahedral cation sites. When Mg is introduced into this already disordered framework, the number of possible configurations increases dramatically due to the variability in choosing Ga sites for substitution, resulting in a high configurational entropy. This entropy contribution lowers the free energy of the γ phase relative to the other more ordered phases. Figure 6 shows relative finite-temperature stability of epitaxially strained β and γ phases including these effects, as described in the Computational Details section above. While in this calculation, the β phase still remains the most stable at both the lowest and highest temperatures shown (which correspond to the lowest and highest temperatures from Figure 1), we note that at higher temperatures, this difference is less than *k*_B_ *T* (as indicated by shaded bands). This is consistent with our proposed explanation for the “alternation” of phases observed in Figure 1: In the initial growth, kinetic effects stabilize the γ phase. At the lower temperature, even though there is a thermodynamic drive to transform, there is insufficient thermal energy to overcome the kinetic barrier. At the intermediate temperature, some diffusion occurs from the substrate into the film, helping to stabilize the layer nearest to the substrate, but the outer layer of the film still transforms to β. Finally, at the highest temperature, diffusion is fast enough that the driving force for transformation is sufficiently reduced throughout the film so as to maintain γ throughout the thickness.

**Figure 6:**
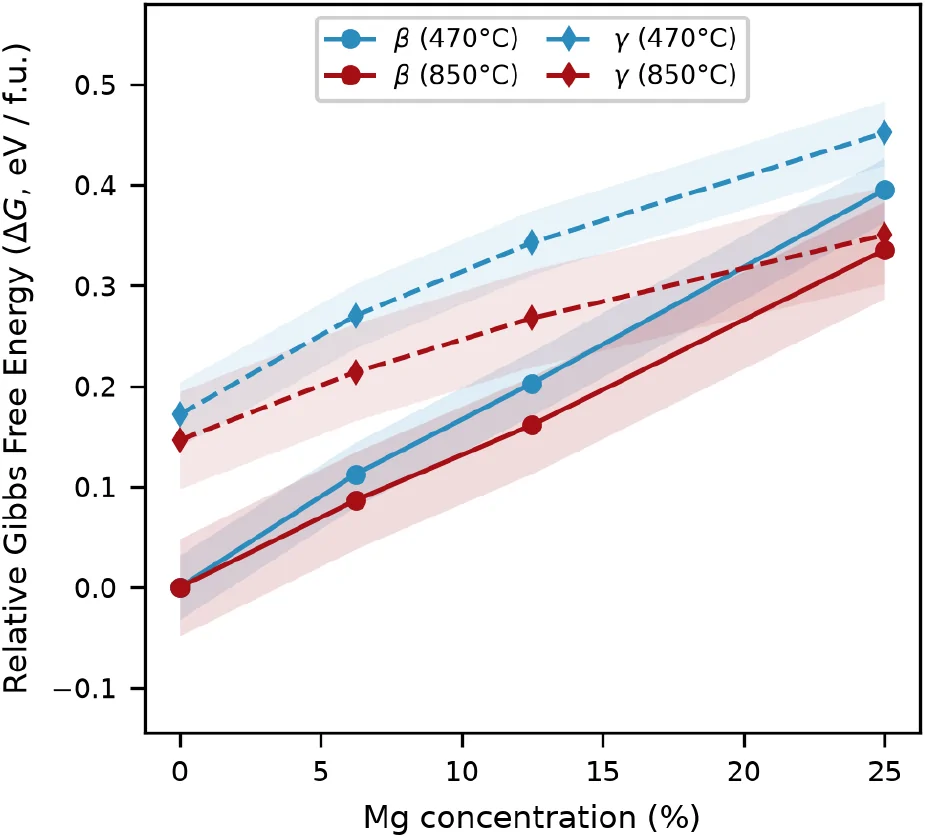
Gibbs free energy curves for epitaxially strained β and γ phases at 470 and 850 °C, calculated with configurational entropy contributions for the *TS* terms and DFT energies for the enthalpies. Energies are referenced to pure β-Ga_2_O_3_. Shaded bands have width *k*_B_ *T*.

We note also that in this work, we have assumed that the γ phase remains “Ga_2_O_3_-like”—namely, that the cation:oxygen ratio remains approximately 2:3, with all deviations arising due to the additional vacancies introduced to preserve charge balance under Mg incorporation. In reality, experimental characterization suggests that at higher Mg and Al incorporation, these films may be better described as spinel-structured solid solutions. Future work may explore the broader spectrum of disorder that this implies.

## Conclusion

In this work, we investigated the effect of Mg substitution on the relative stability of Ga_2_O_3_ polymorphs, with a particular focus on the β and γ phases that have been experimentally observed in thin films grown on Mg-containing substrates. Our calculations indicate that Mg incorporation reduces the enthalpy and free energy differences between the different phases of Ga_2_O_3_, thereby lowering the energetic barrier to forming metastable phases. For β-Ga_2_O_3_, Mg exhibits a strong preference for octahedral Ga sites, and this preference becomes increasingly significant at higher Mg incorporation, where octahedral substitution substantially reduces the enthalpy of formation relative to other sites. In contrast, Mg shows no clear site preference in γ-Ga_2_O_3_, consistent with the structural disorder of this phase. When considered alongside prior work, these results provide a mechanism for the experimentally observed stabilization of γ-Ga_2_O_3_ on Mg-containing substrates. More broadly, the results highlight cation substitution as a practical route to tailoring polymorph stability in Ga_2_O_3_ semiconductors.

## Supplementary Material



## Data Availability

Input structures, preprocessing code, and simulation input files are freely available on GitHub at https://github.com/ACME-group-CMU/Ga2O3_Mg_paper.

## Notes

More detailed description of the process; selection of representative sets of alloy structures; associated figures (PDF)
