# Supplementary material for: First-Principles Study of Magnesium-Induced Phase Stabilization in Gallium Oxide Polymorphs
Source: Chem Mater. 2026 Aug 4;38(16):8657–64. doi: 10.1021/acs.chemmater.6c01704 (PMC13523635; doi:10.1021/acs.chemmater.6c01704)
Supplement: Supplementary file 1 [file cm6c01704_si_001.pdf]

First-principles study of magnesium-induced phase stabilization in gallium oxide polymorphs: Supporting Information

Viswesh Prakash, Jingyu Tang, Lisa M. Porter, and Rachel C. Kurchin*

Department of Materials Science and Engineering, Carnegie Mellon University, Pittsburgh, Pennsylvania 15213, USA

E-mail: rkurchin@cmu.edu

Selection of Representative Subset of Alloy Structures

For each structure, we compute the normalized partial radial distribution functions for all distinct atomic pair combinations: Ga–Ga, Ga–Mg, Ga–O, Ga–vacancy, Mg–Mg, Mg–O, Mg–vacancy, O–O, O–vacancy, and vacancy–vacancy. These RDFs provide a quantitative description of the local atomic environments and spatial correlations within the structure.[1]

We then calculate the average RDF for each pair type across the entire pool of generated structures, establishing a reference for the overall distribution of local environments. Subsequently, multiple random subsets of structures are drawn for a given sample size, and the average RDFs for each subset are computed. An example of the comparison between subset averages and the full-set average for all pairwise RDFs of γ-Ga_2O_3 supercell with 25% of Ga sites replaced at random with Mg is shown in Figure S1.

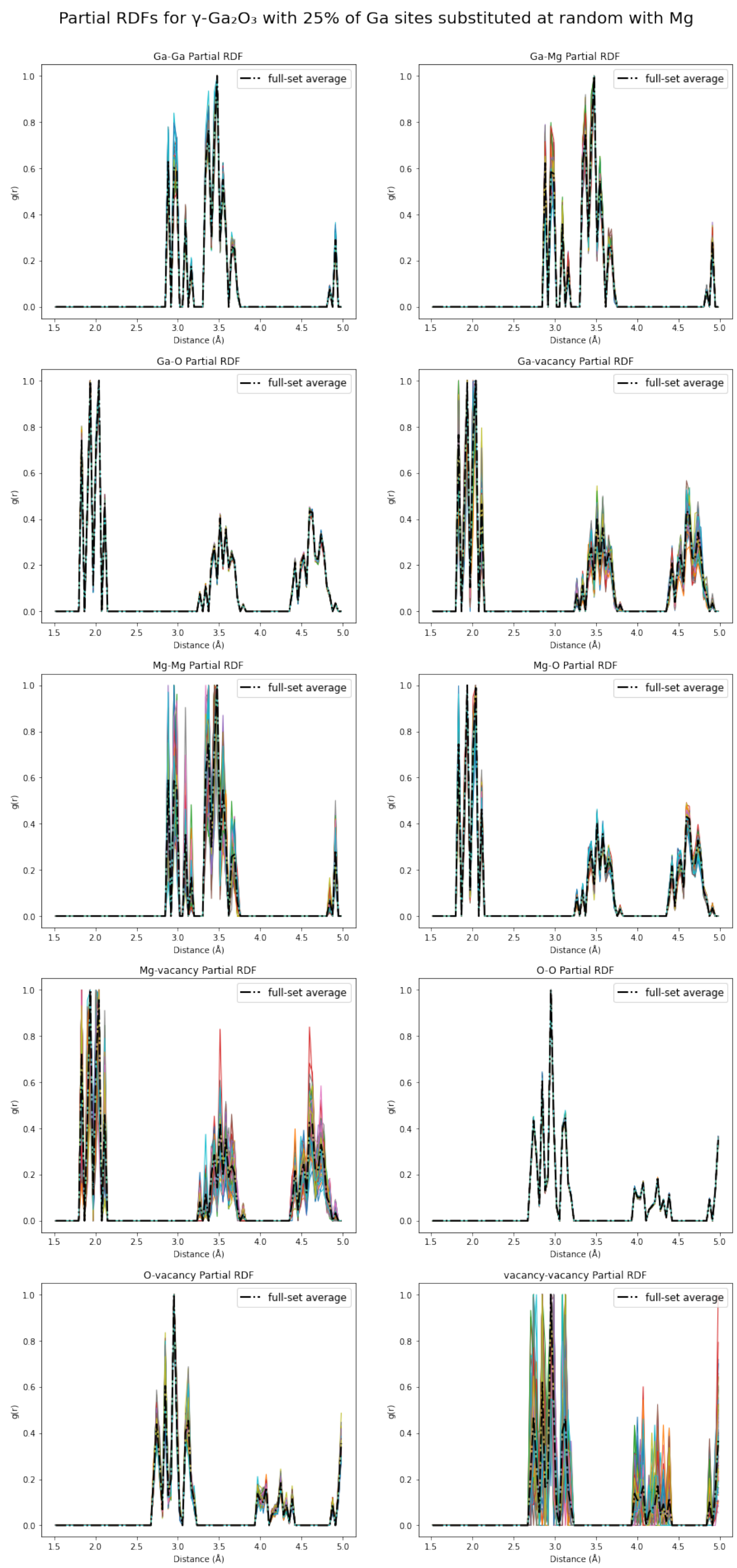

Figure S1: Comparison of average normalized partial radial distribution functions for 100 randomly selected subsets of 10 structures (shown in solid colored lines) with the full-set average from 1000 symmetrically inequivalent structures (dot-dash black line) for γ-Ga_2O_3 with 25% of Ga sites randomly substituted by Mg. The RDFs include all relevant atomic pair combinations (Ga–Ga, Ga–Mg, Ga–O, Ga–vacancy, Mg–Mg, Mg–O, Mg–vacancy, O–O, O–vacancy, and vacancy–vacancy). Note: Only distances (x-axis) greater than 1.5 Å are shown for given plots, RDF probabilities are zero for shorter distances. (See supporting information for RDF distributions for all relevant substitution cases)

To assess the similarity between the subset averages and the full-set average, we use the Kullback–Leibler (KL) divergence.[2] KL divergence provides a measure of dissimilarity between probability distributions, where a value of 0 indicates identical distributions, and higher values reflect increasing divergence. Since KL divergence is asymmetric, we compute the KL divergence from each subset average to the full-set average, which quantifies how well the former approximates the latter. For each subset, KL divergences are calculated for all pairwise RDFs, and a weighted average divergence is computed. The weights correspond to the number of occurrences of each pair type in the structure, ensuring that more frequent interactions contribute proportionally to the overall metric.

The subset exhibiting the lowest weighted KL divergence is selected as the most representative of the full configuration space, as its average local environment closely approximates that of the entire ensemble. This approach ensures the selection of a physically meaningful and computationally tractable subset of structures. We validated this sampling methodology across multiple sample sizes (see Figure S2) and found that a subset size of ten structures provided an optimal balance between computational efficiency and representational accuracy. The resulting subsets consistently show KL divergence values comparable to larger samples, confirming their statistical adequacy for further first-principles analysis.

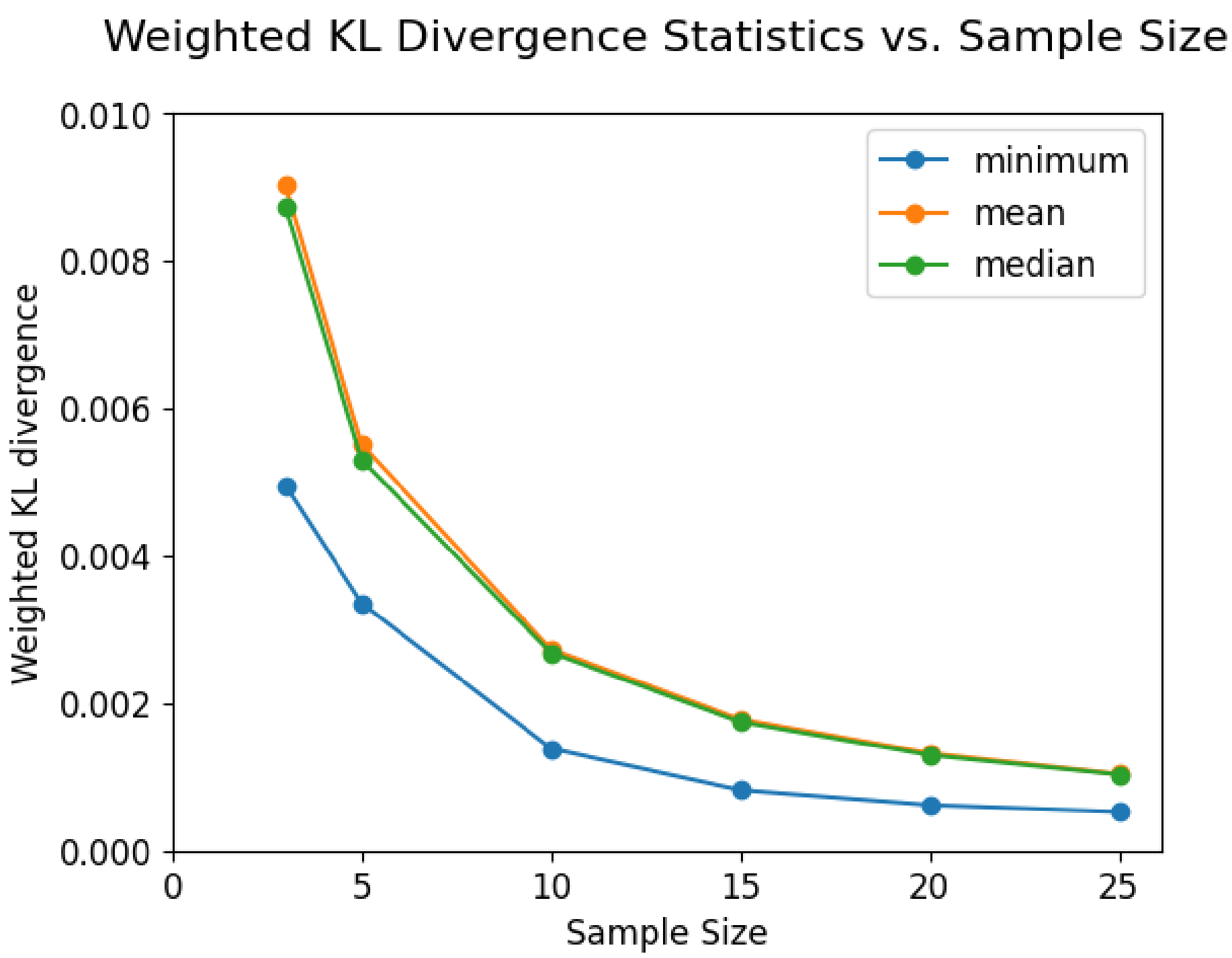

Figure S2: Plot showing the minimum, mean, and median values of the weighted Kullback–Leibler (KL) divergence across 100 randomly selected subsets of symmetrically inequivalent γ-Ga_2O_3 structures with 25% Mg substitution, shown as a function of sample size. The convergence of all three metrics at sample size of 10 demonstrates that it provides a representative approximation of the full distribution, justifying its use for further calculations.

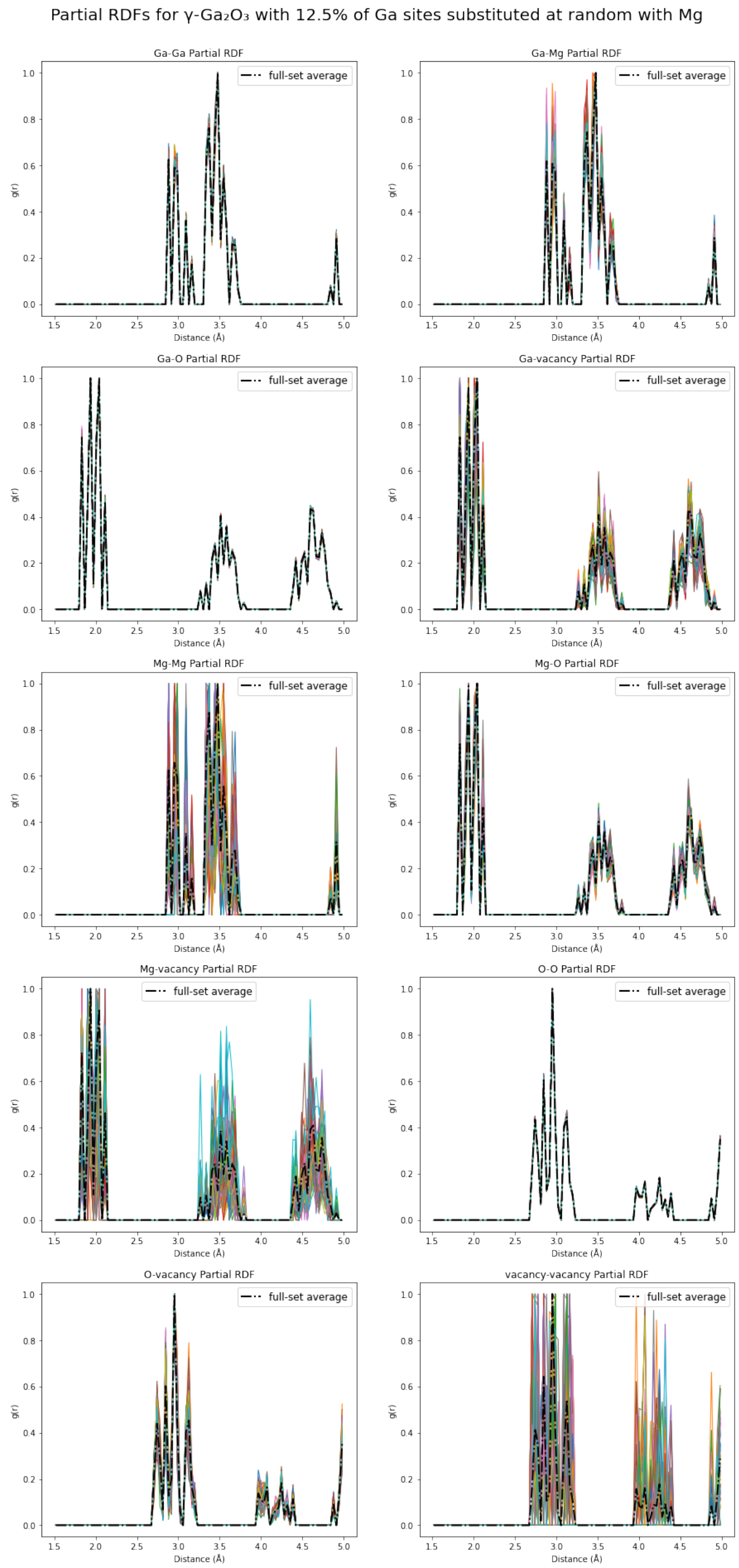

Figure S3: Comparison of average normalized partial radial distribution functions for 100 randomly selected subsets of 10 structures (shown in solid colored lines) with the full-set average from 1000 symmetrically inequivalent structures (dot-dash black line) for γ-Ga_2O_3 with 12.5% of Ga sites randomly substituted by Mg. The RDFs include all relevant atomic pair combinations (Ga–Ga, Ga–Mg, Ga–O, Ga–vacancy, Mg–Mg, Mg–O, Mg–vacancy, O–O, O–vacancy, and vacancy–vacancy). Note: Only distances (x-axis) greater than 1.5 Å are shown for given plots, RDF probabilities are zero for shorter distances.

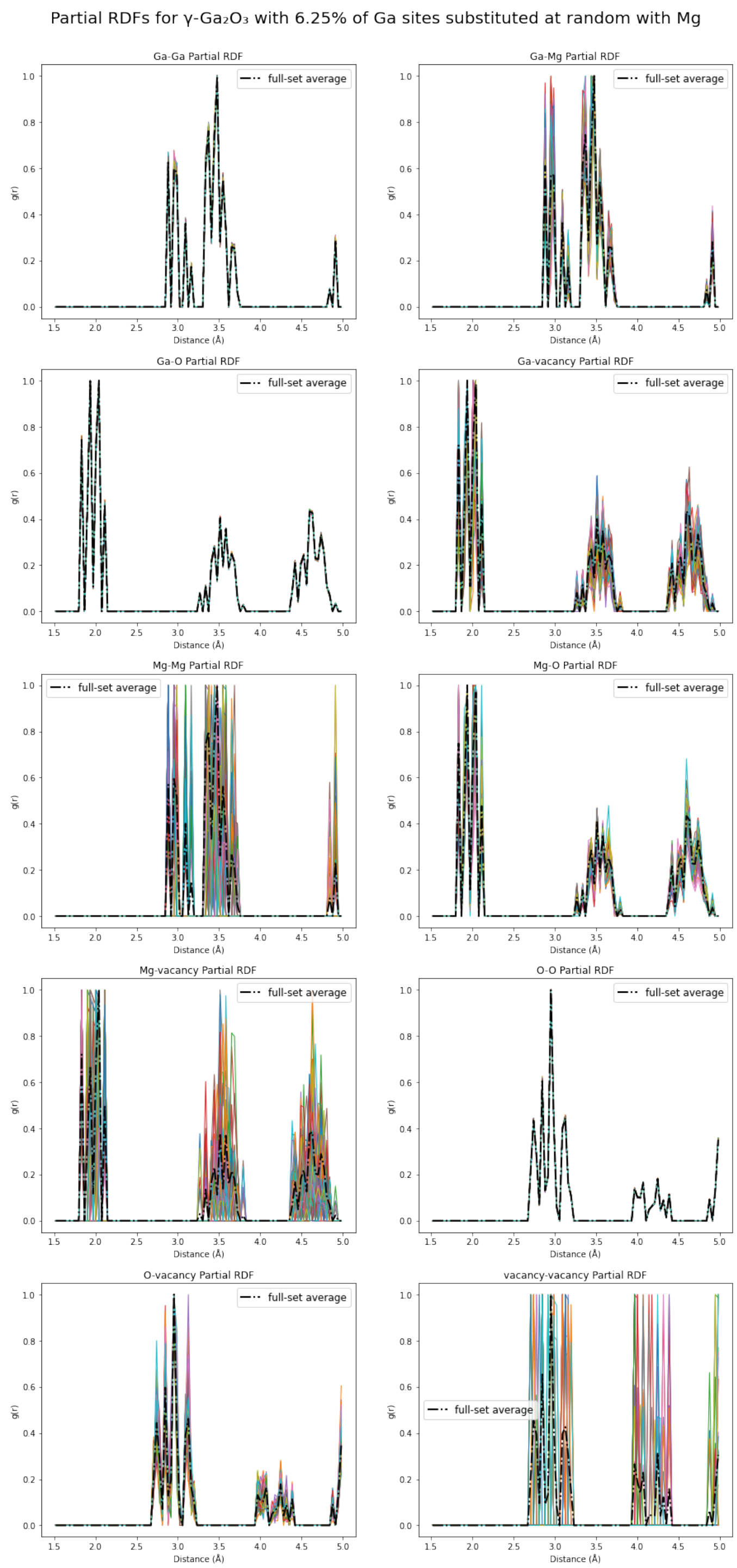

Figure S4: Comparison of average normalized partial radial distribution functions for 100 randomly selected subsets of 10 structures (shown in solid colored lines) with the full-set average from 1000 symmetrically inequivalent structures (dot-dash black line) for γ-Ga_2O_3 with 6.25% of Ga sites randomly substituted by Mg. The RDFs include all relevant atomic pair combinations (Ga–Ga, Ga–Mg, Ga–O, Ga–vacancy, Mg–Mg, Mg–O, Mg–vacancy, O–O, O–vacancy, and vacancy–vacancy). Note: Only distances (x-axis) greater than 1.5 Å are shown for given plots, RDF probabilities are zero for shorter distances.

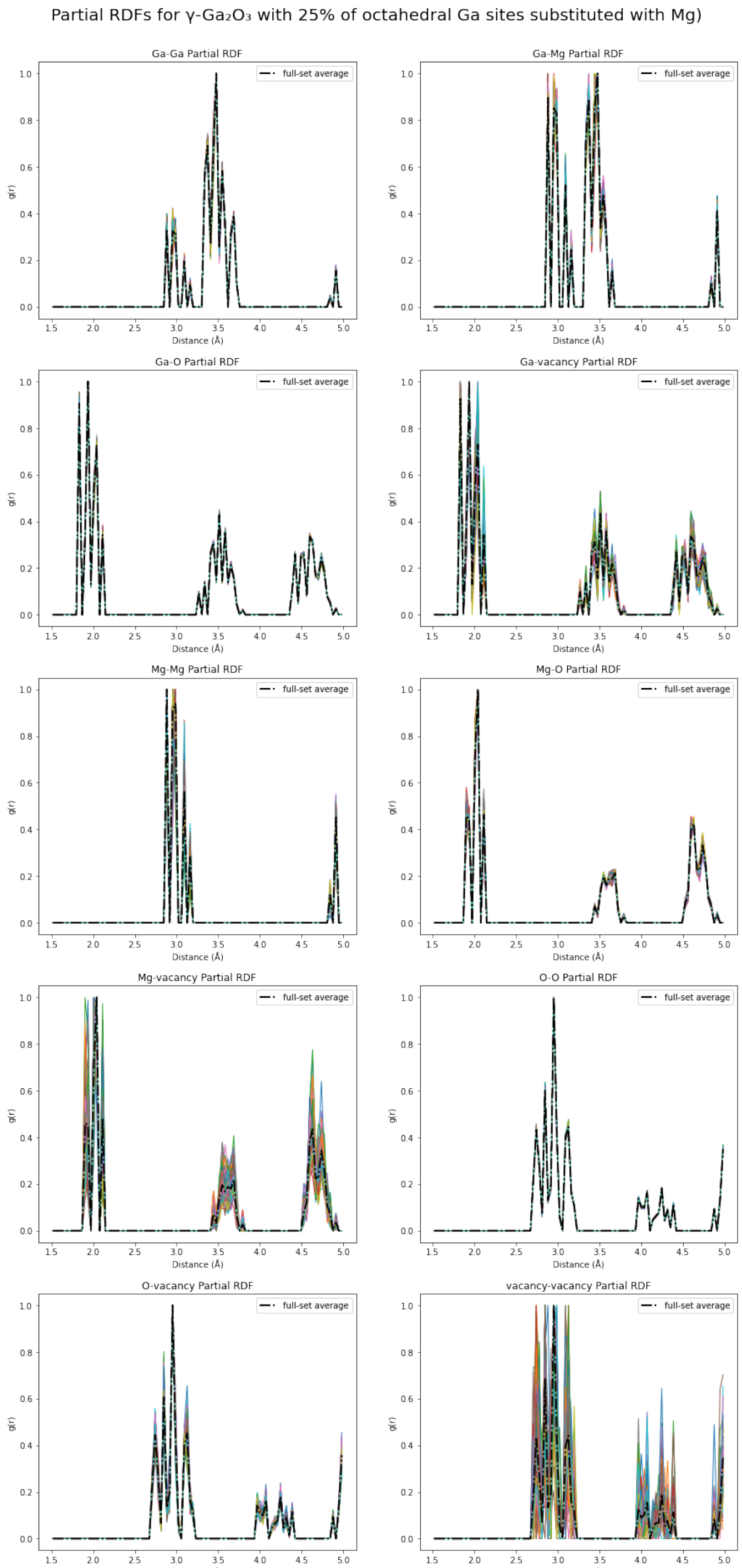

Figure S5: Comparison of average normalized partial radial distribution functions for 100 randomly selected subsets of 10 structures (shown in solid colored lines) with the full-set average from 1000 symmetrically inequivalent structures (dot-dash black line) for γ-Ga_2O_3 with 25% of octahedral Ga sites substituted by Mg. The RDFs include all relevant atomic pair combinations (Ga–Ga, Ga–Mg, Ga–O, Ga–vacancy, Mg–Mg, Mg–O, Mg–vacancy, O–O, O–vacancy, and vacancy–vacancy). Note: Only distances (x-axis) greater than 1.5 Å are shown for given plots, RDF probabilities are zero for shorter distances.

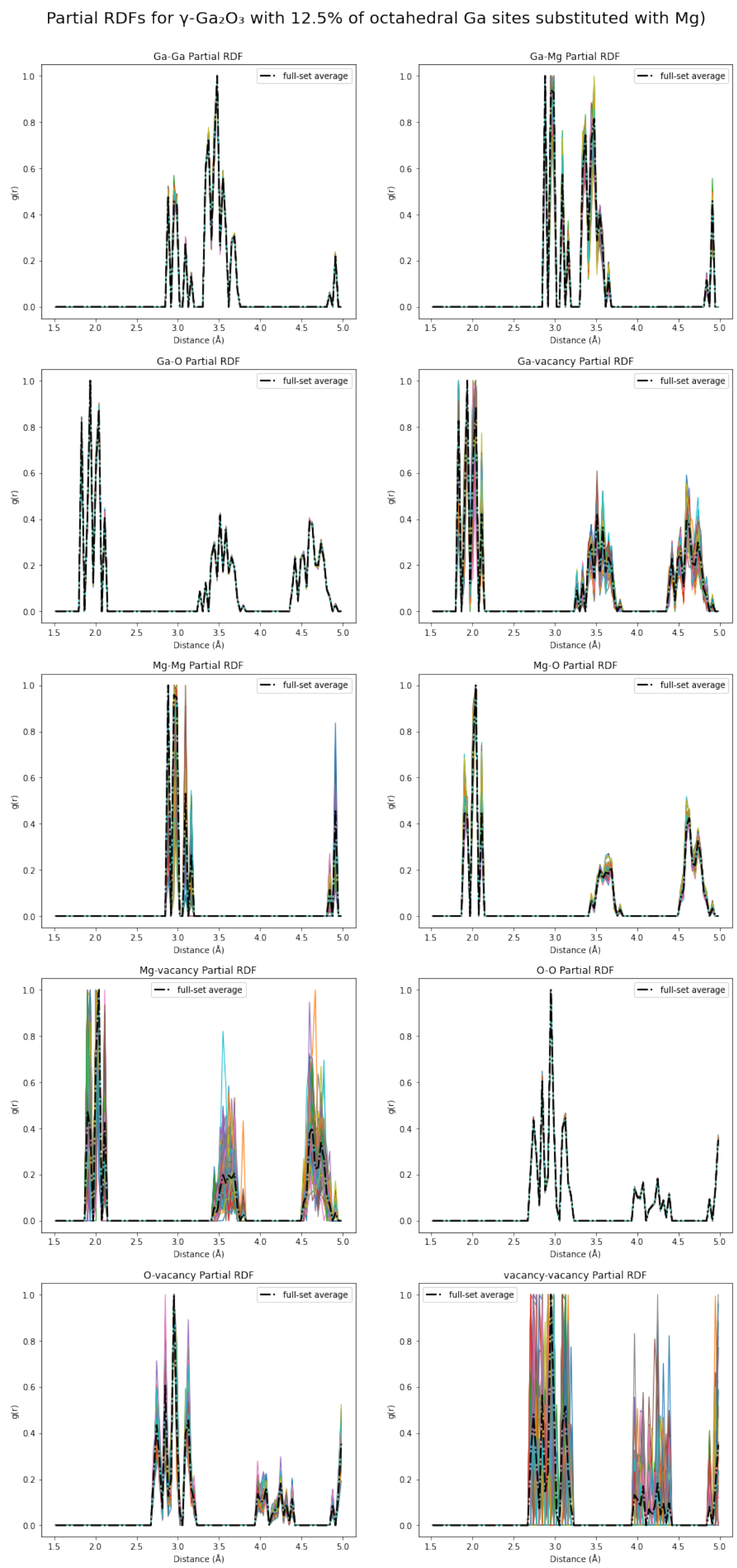

Figure S6: Comparison of average normalized partial radial distribution functions for 100 randomly selected subsets of 10 structures (shown in solid colored lines) with the full-set average from 1000 symmetrically inequivalent structures (dot-dash black line) for γ-Ga_2O_3 with 12.5% of octahedral Ga sites substituted by Mg. The RDFs include all relevant atomic pair combinations (Ga–Ga, Ga–Mg, Ga–O, Ga–vacancy, Mg–Mg, Mg–O, Mg–vacancy, O–O, O–vacancy, and vacancy–vacancy). Note: Only distances (x-axis) greater than 1.5 Å are shown for given plots, RDF probabilities are zero for shorter distances.

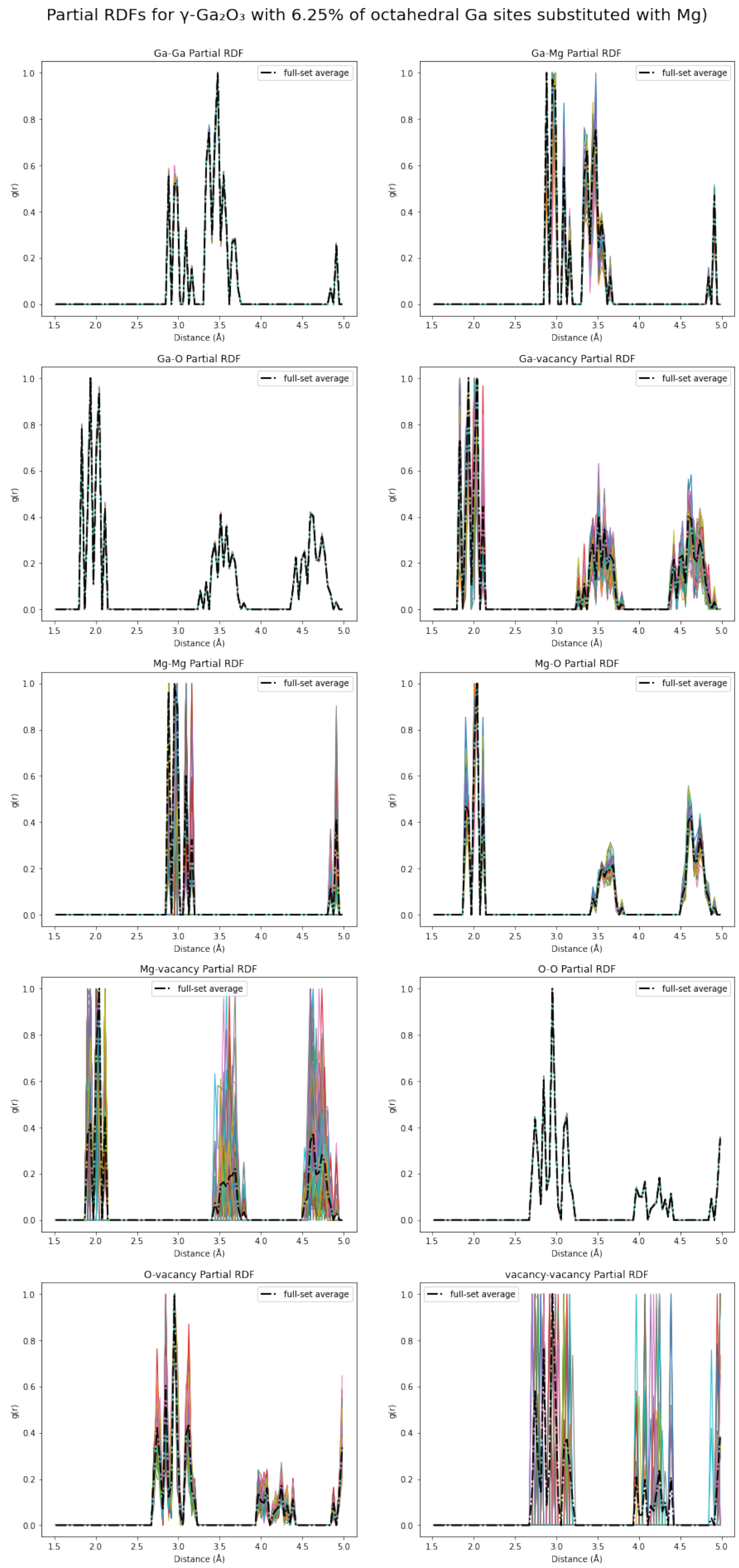

Figure S7: Comparison of average normalized partial radial distribution functions for 100 randomly selected subsets of 10 structures (shown in solid colored lines) with the full-set average from 1000 symmetrically inequivalent structures (dot-dash black line) for γ-Ga_2O_3 with 6.25% of octahedral Ga sites substituted by Mg. The RDFs include all relevant atomic pair combinations (Ga–Ga, Ga–Mg, Ga–O, Ga–vacancy, Mg–Mg, Mg–O, Mg–vacancy, O–O, O–vacancy, and vacancy–vacancy). Note: Only distances (x-axis) greater than 1.5 Å are shown for given plots, RDF probabilities are zero for shorter distances.

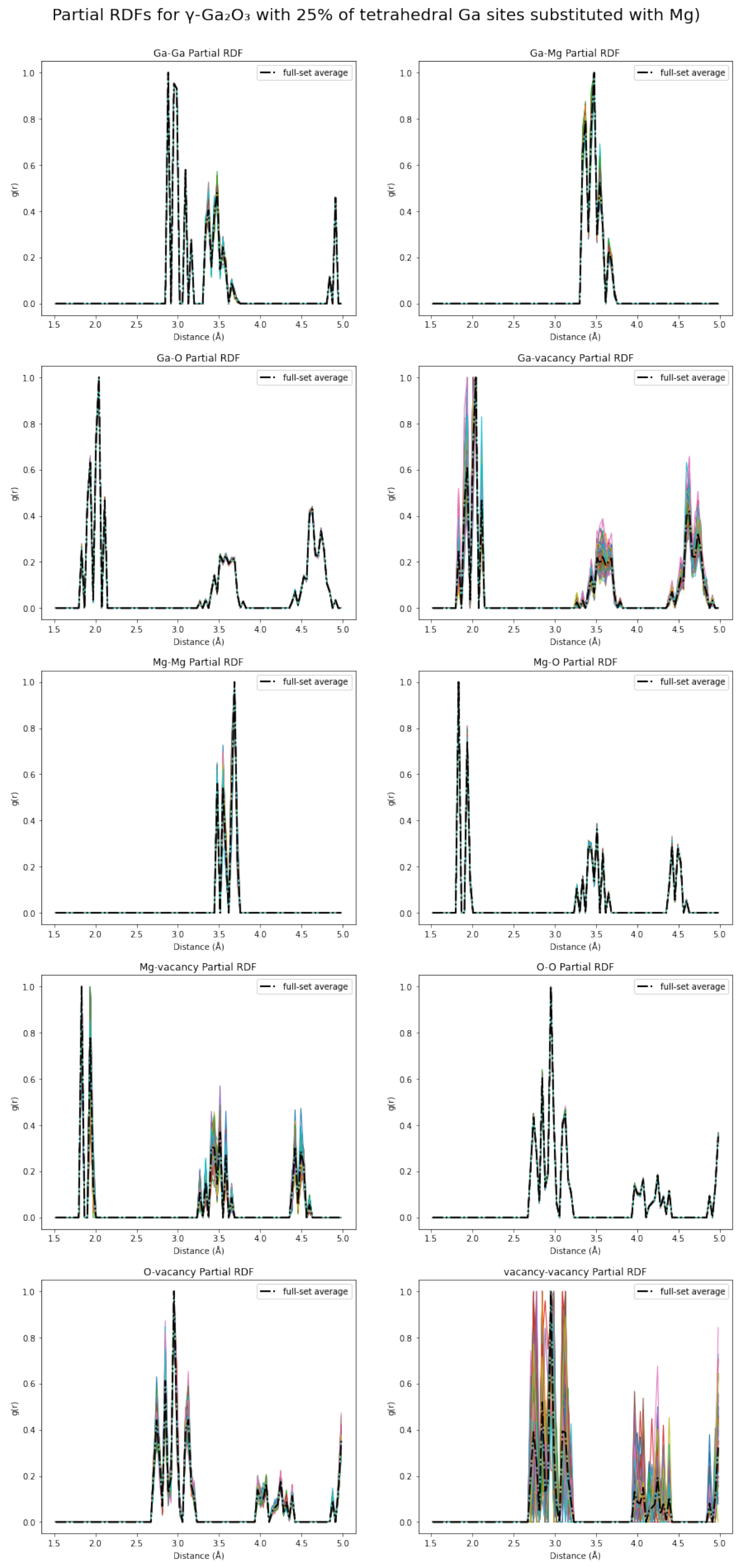

Figure S8: Comparison of average normalized partial radial distribution functions for 100 randomly selected subsets of 10 structures (shown in solid colored lines) with the full-set average from 1000 symmetrically inequivalent structures (dot-dash black line) for γ-Ga_2O_3 with 25% of tetrahedral Ga sites substituted by Mg. The RDFs include all relevant atomic pair combinations (Ga–Ga, Ga–Mg, Ga–O, Ga–vacancy, Mg–Mg, Mg–O, Mg–vacancy, O–O, O–vacancy, and vacancy–vacancy). Note: Only distances (x-axis) greater than 1.5 Å are shown for given plots, RDF probabilities are zero for shorter distances.

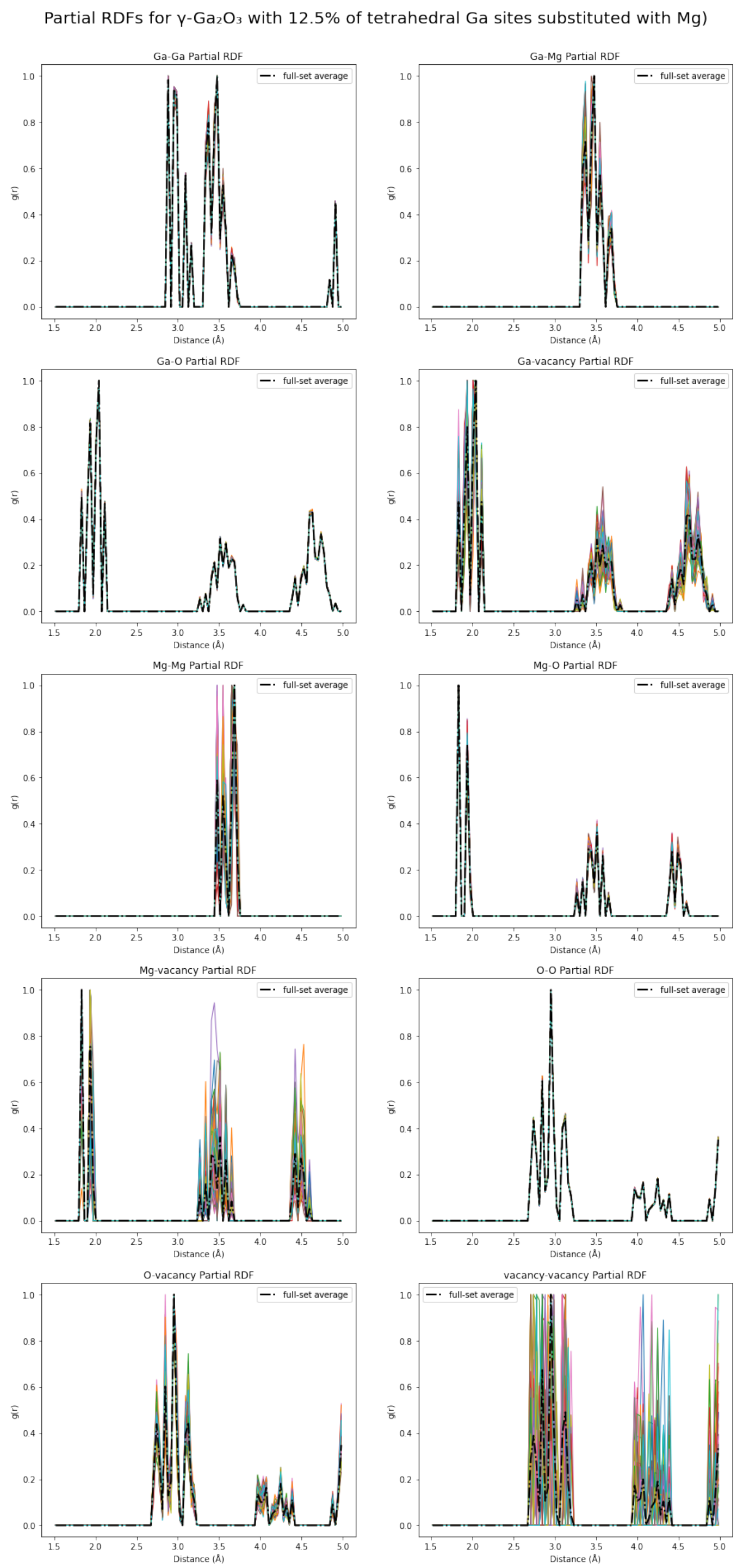

Figure S9: Comparison of average normalized partial radial distribution functions for 100 randomly selected subsets of 10 structures (shown in solid colored lines) with the full-set average from 1000 symmetrically inequivalent structures (dot-dash black line) for γ-Ga_2O_3 with 12.5% of tetrahedral Ga sites substituted by Mg. The RDFs include all relevant atomic pair combinations (Ga–Ga, Ga–Mg, Ga–O, Ga–vacancy, Mg–Mg, Mg–O, Mg–vacancy, O–O, O–vacancy, and vacancy–vacancy). Note: Only distances (x-axis) greater than 1.5 Å are shown for given plots, RDF probabilities are zero for shorter distances.

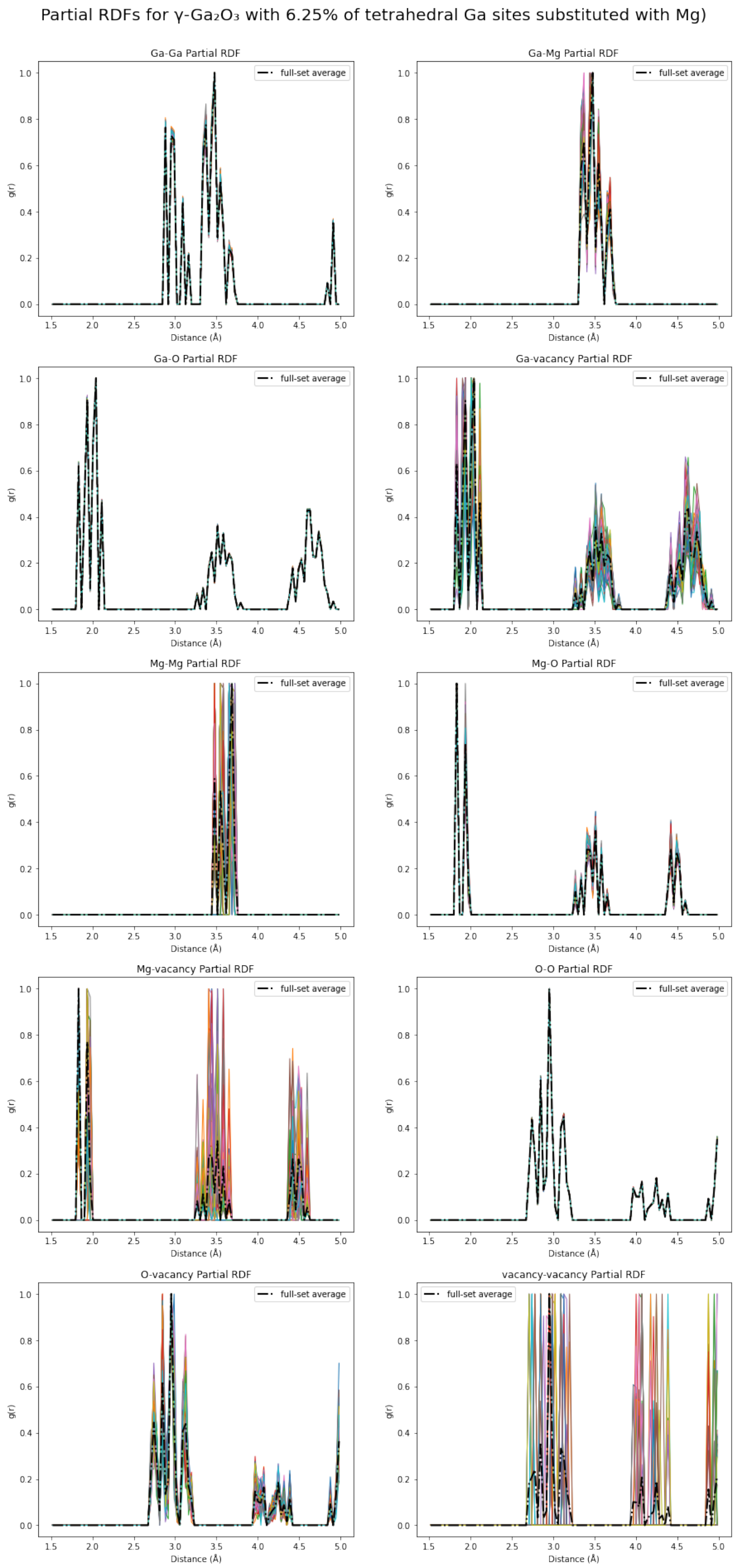

Figure S10: Comparison of average normalized partial radial distribution functions for 100 randomly selected subsets of 10 structures (shown in solid colored lines) with the full-set average from 1000 symmetrically inequivalent structures (dot-dash black line) for γ-Ga_2O_3 with 6.25% of tetrahedral Ga sites substituted by Mg. The RDFs include all relevant atomic pair combinations (Ga–Ga, Ga–Mg, Ga–O, Ga–vacancy, Mg–Mg, Mg–O, Mg–vacancy, O–O, O–vacancy, and vacancy–vacancy). Note: Only distances (x-axis) greater than 1.5 Å are shown for given plots, RDF probabilities are zero for shorter distances.

Comparing Polymorphs Across Substitution Sites

Figure S20 presents an alternate visualization of the same data from Figure 3 in the main text that more easily enables comparison of polymorphs within the same substitution site rather than substitution sites within the same polymorph.

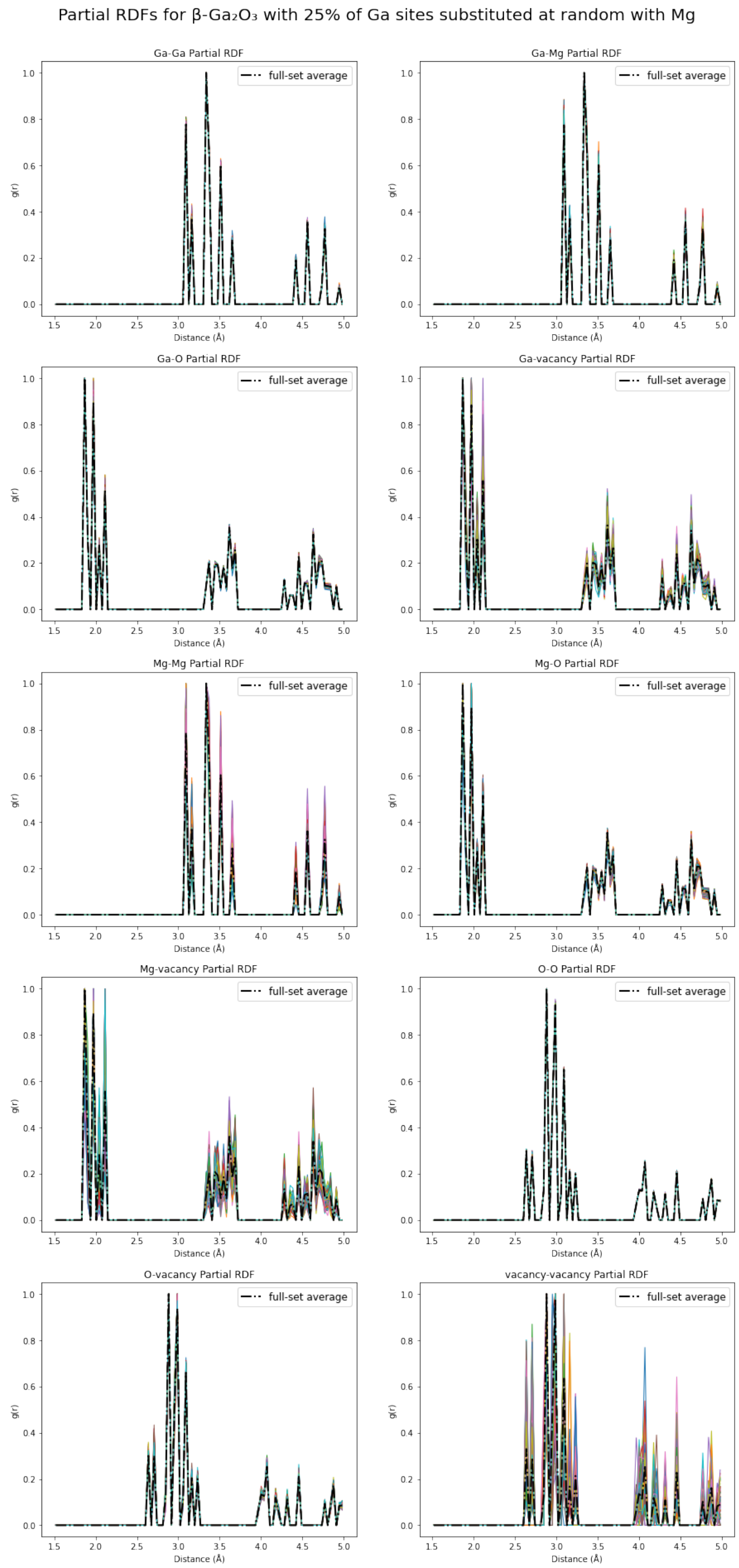

Figure S11: Comparison of average normalized partial radial distribution functions for 100 randomly selected subsets of 10 structures (shown in solid colored lines) with the full-set average from 1000 symmetrically inequivalent structures (dot-dash black line) for β-Ga_2O_3 with 25% of Ga sites randomly substituted by Mg. The RDFs include all relevant atomic pair combinations (Ga–Ga, Ga–Mg, Ga–O, Ga–vacancy, Mg–Mg, Mg–O, Mg–vacancy, O–O, O–vacancy, and vacancy–vacancy). Note: Only distances (x-axis) greater than 1.5 Å are shown for given plots, RDF probabilities are zero for shorter distances.

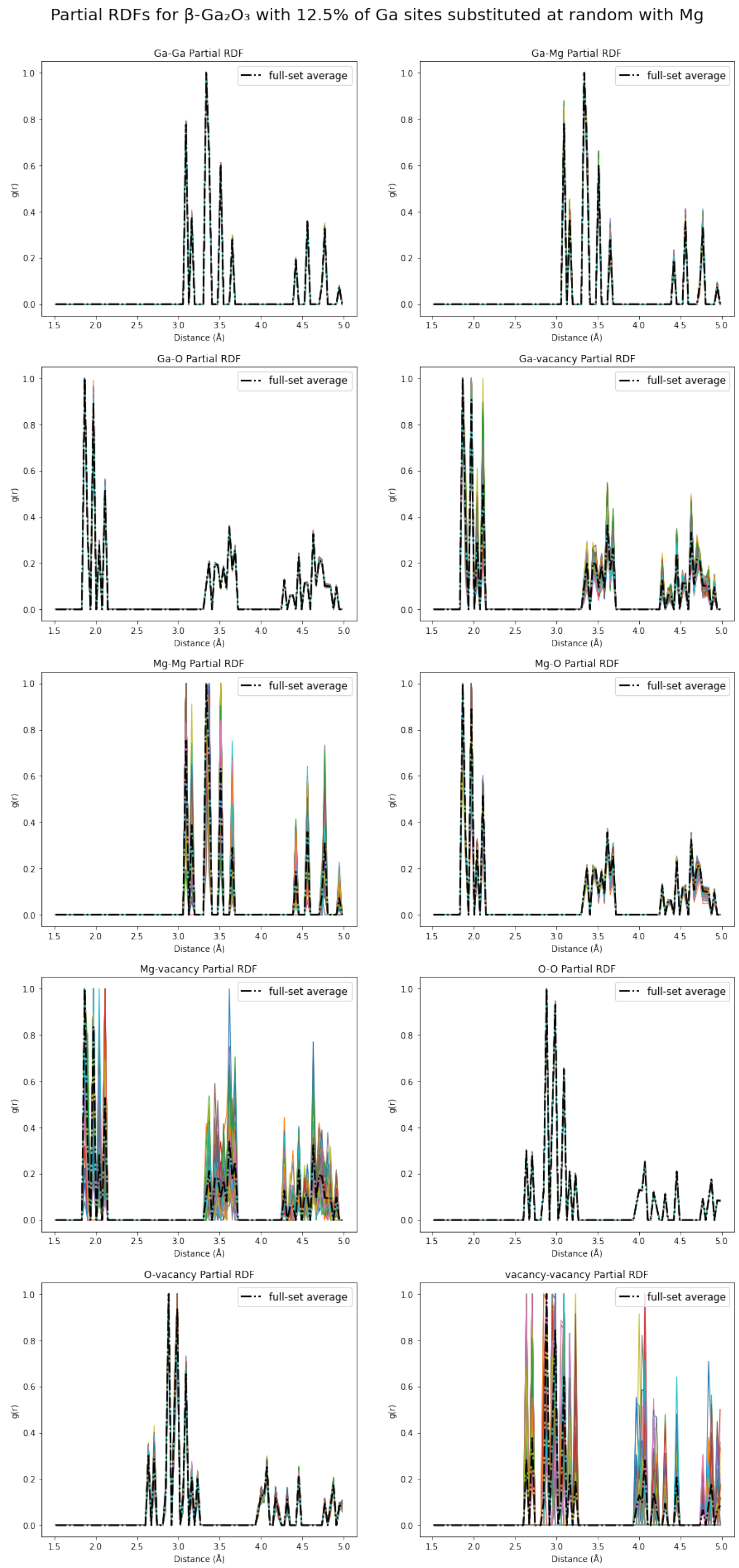

Figure S12: Comparison of average normalized partial radial distribution functions for 100 randomly selected subsets of 10 structures (shown in solid colored lines) with the full-set average from 1000 symmetrically inequivalent structures (dot-dash black line) for β-Ga_2O_3 with 12.5% of Ga sites randomly substituted by Mg. The RDFs include all relevant atomic pair combinations (Ga–Ga, Ga–Mg, Ga–O, Ga–vacancy, Mg–Mg, Mg–O, Mg–vacancy, O–O, O–vacancy, and vacancy–vacancy). Note: Only distances (x-axis) greater than 1.5 Å are shown for given plots, RDF probabilities are zero for shorter distances.

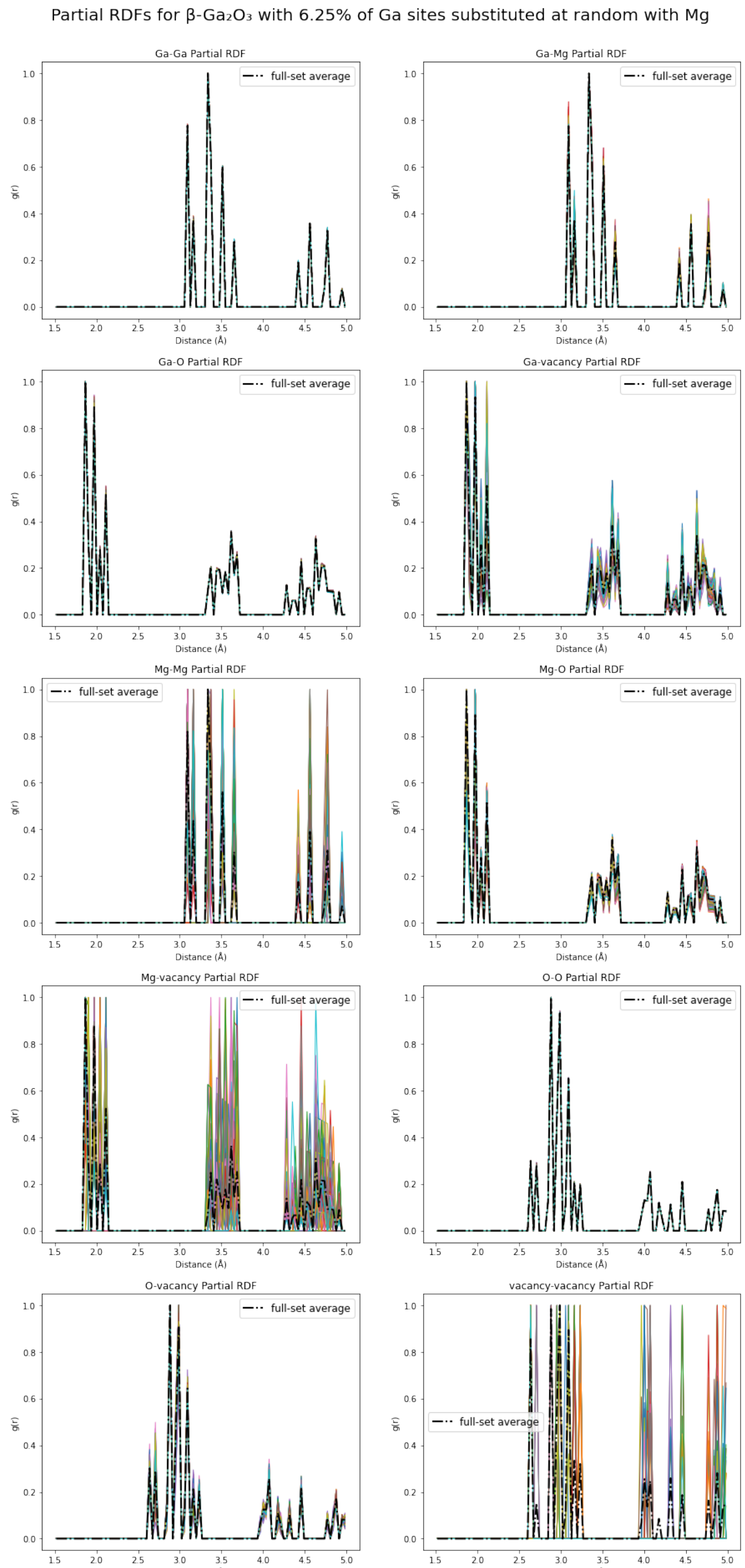

Figure S13: Comparison of average normalized partial radial distribution functions for 100 randomly selected subsets of 10 structures (shown in solid colored lines) with the full-set average from 1000 symmetrically inequivalent structures (dot-dash black line) for β-Ga_2O_3 with 6.25% of Ga sites randomly substituted by Mg. The RDFs include all relevant atomic pair combinations (Ga–Ga, Ga–Mg, Ga–O, Ga–vacancy, Mg–Mg, Mg–O, Mg–vacancy, O–O, O–vacancy, and vacancy–vacancy). Note: Only distances (x-axis) greater than 1.5 Å are shown for given plots, RDF probabilities are zero for shorter distances.

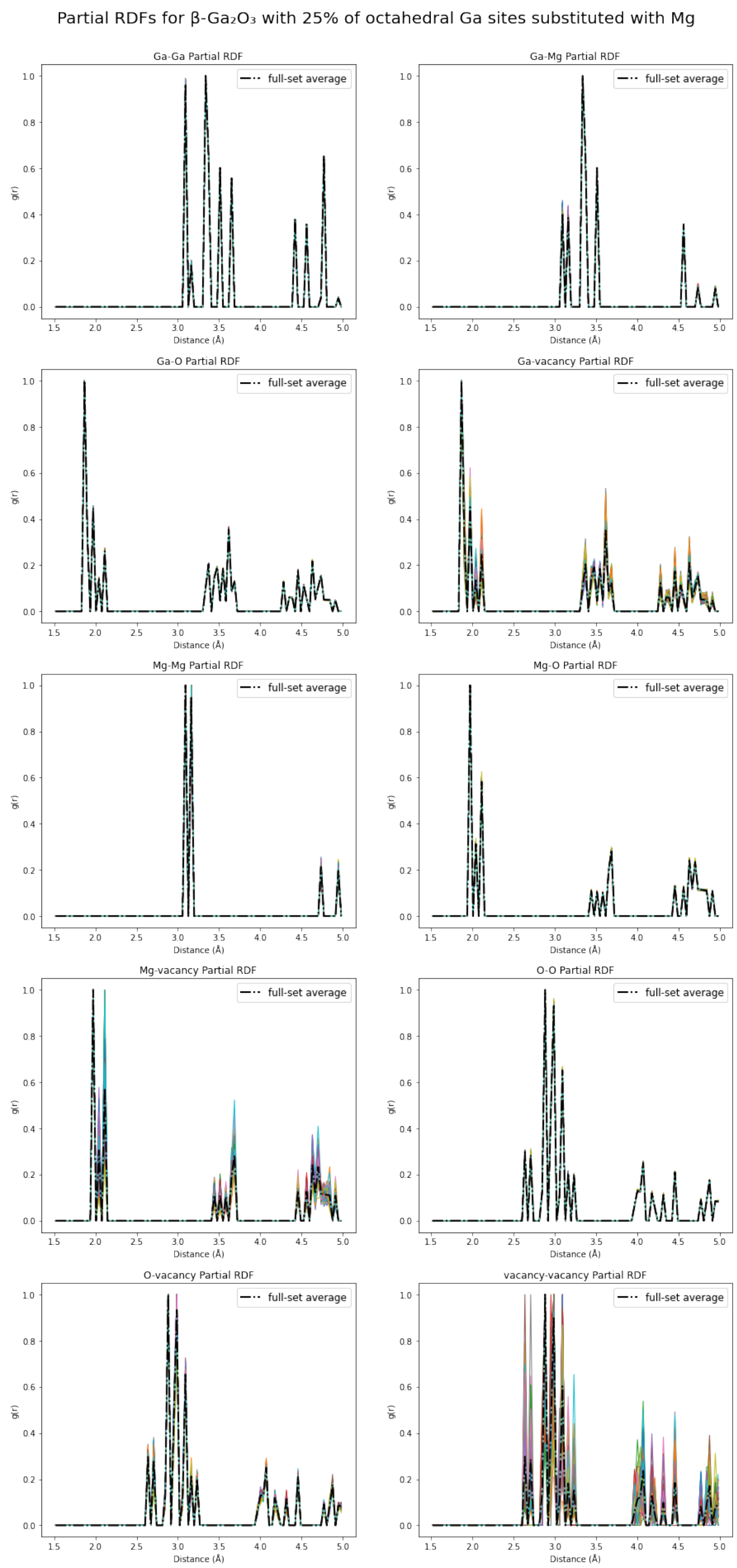

Figure S14: Comparison of average normalized partial radial distribution functions for 100 randomly selected subsets of 10 structures (shown in solid colored lines) with the full-set average from 1000 symmetrically inequivalent structures (dot-dash black line) for β-Ga_2O_3 with 25% of octahedral Ga sites substituted by Mg. The RDFs include all relevant atomic pair combinations (Ga–Ga, Ga–Mg, Ga–O, Ga–vacancy, Mg–Mg, Mg–O, Mg–vacancy, O–O, O–vacancy, and vacancy–vacancy). Note: Only distances (x-axis) greater than 1.5 Å are shown for given plots, RDF probabilities are zero for shorter distances.

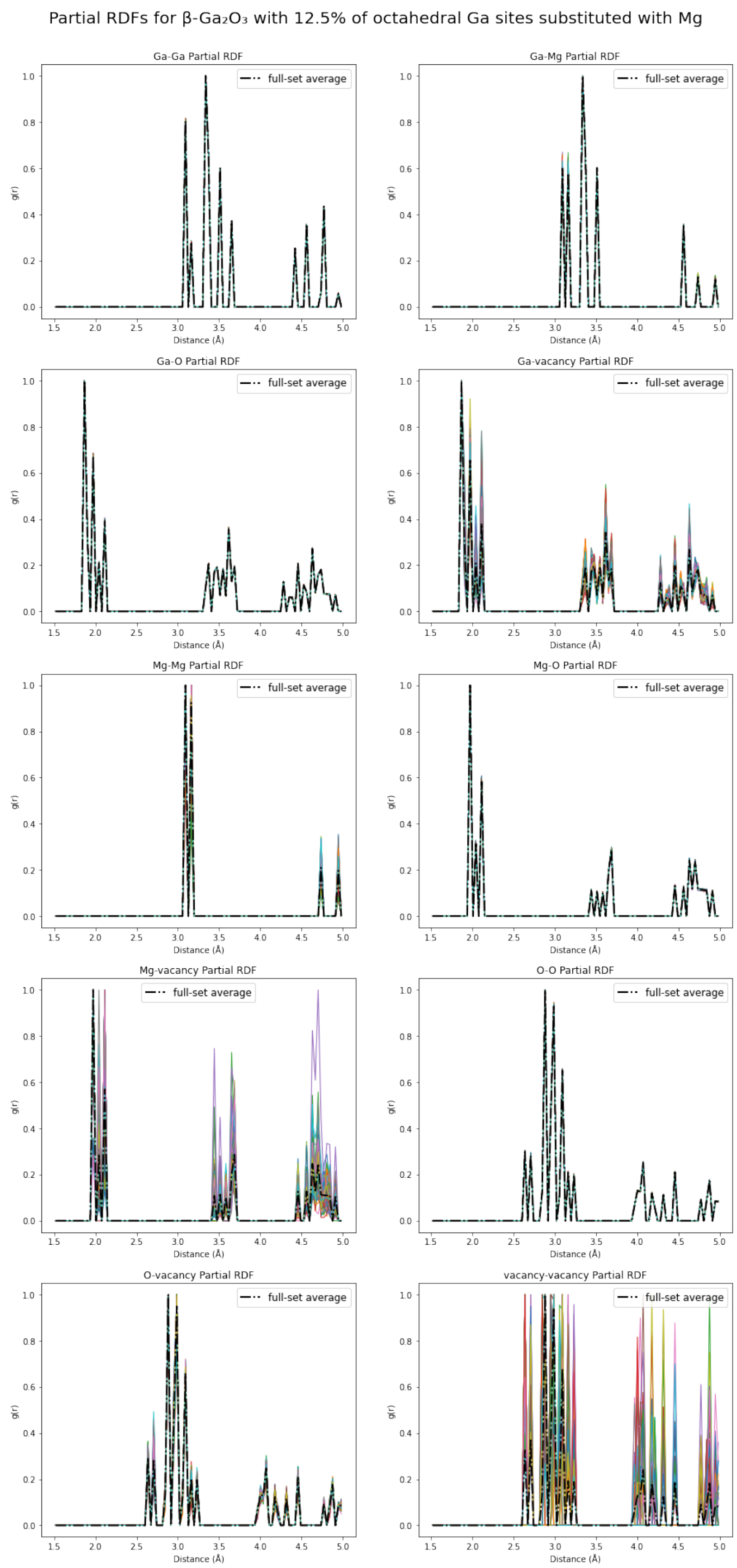

Figure S15: Comparison of average normalized partial radial distribution functions for 100 randomly selected subsets of 10 structures (shown in solid colored lines) with the full-set average from 1000 symmetrically inequivalent structures (dot-dash black line) for β-Ga_2O_3 with 12.5% of octahedral Ga sites substituted by Mg. The RDFs include all relevant atomic pair combinations (Ga–Ga, Ga–Mg, Ga–O, Ga–vacancy, Mg–Mg, Mg–O, Mg–vacancy, O–O, O–vacancy, and vacancy–vacancy). Note: Only distances (x-axis) greater than 1.5 Å are shown for given plots, RDF probabilities are zero for shorter distances.

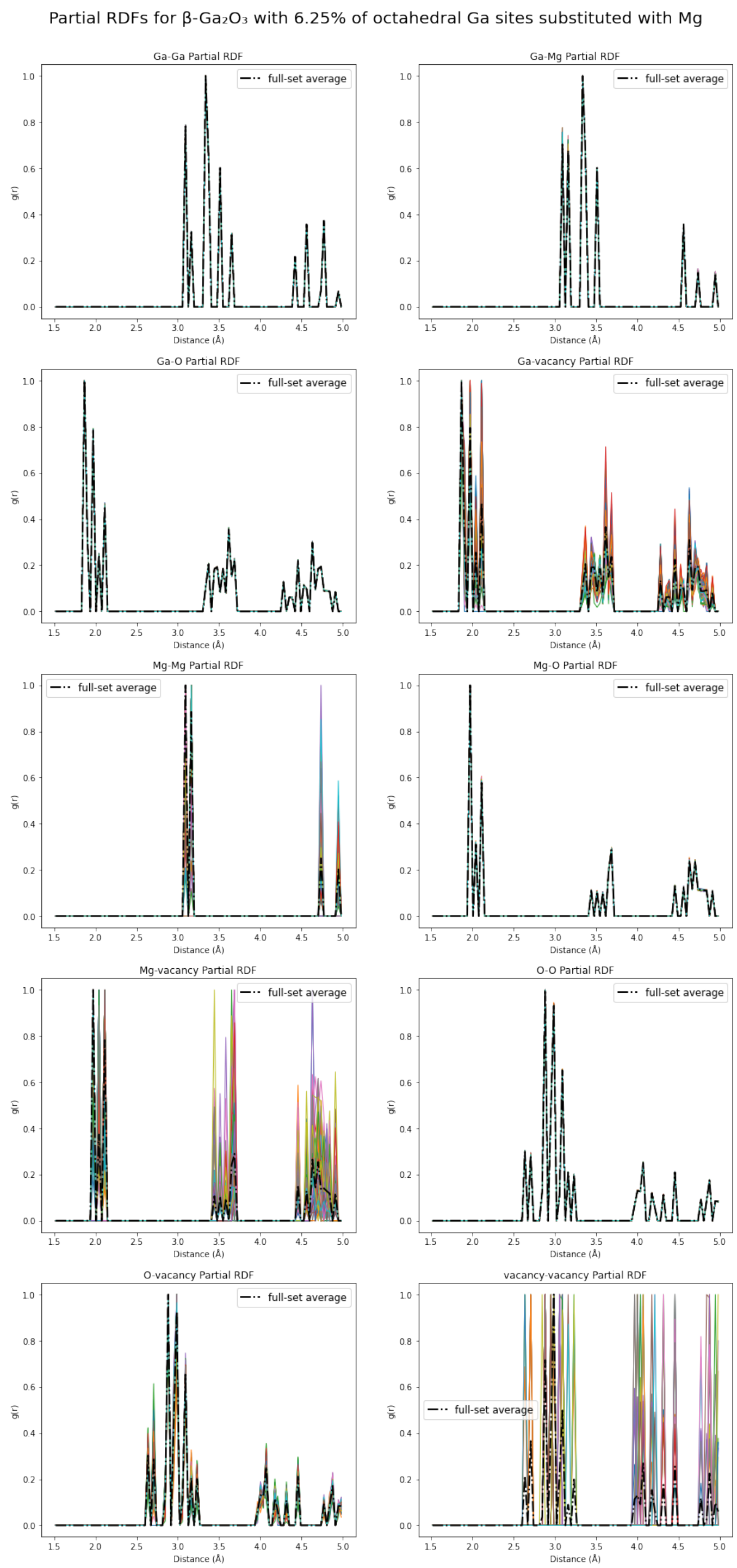

Figure S16: Comparison of average normalized partial radial distribution functions for 100 randomly selected subsets of 10 structures (shown in solid colored lines) with the full-set average from 1000 symmetrically inequivalent structures (dot-dash black line) for β-Ga_2O_3 with 6.25% of octahedral Ga sites substituted by Mg. The RDFs include all relevant atomic pair combinations (Ga–Ga, Ga–Mg, Ga–O, Ga–vacancy, Mg–Mg, Mg–O, Mg–vacancy, O–O, O–vacancy, and vacancy–vacancy). Note: Only distances (x-axis) greater than 1.5 Å are shown for given plots, RDF probabilities are zero for shorter distances.

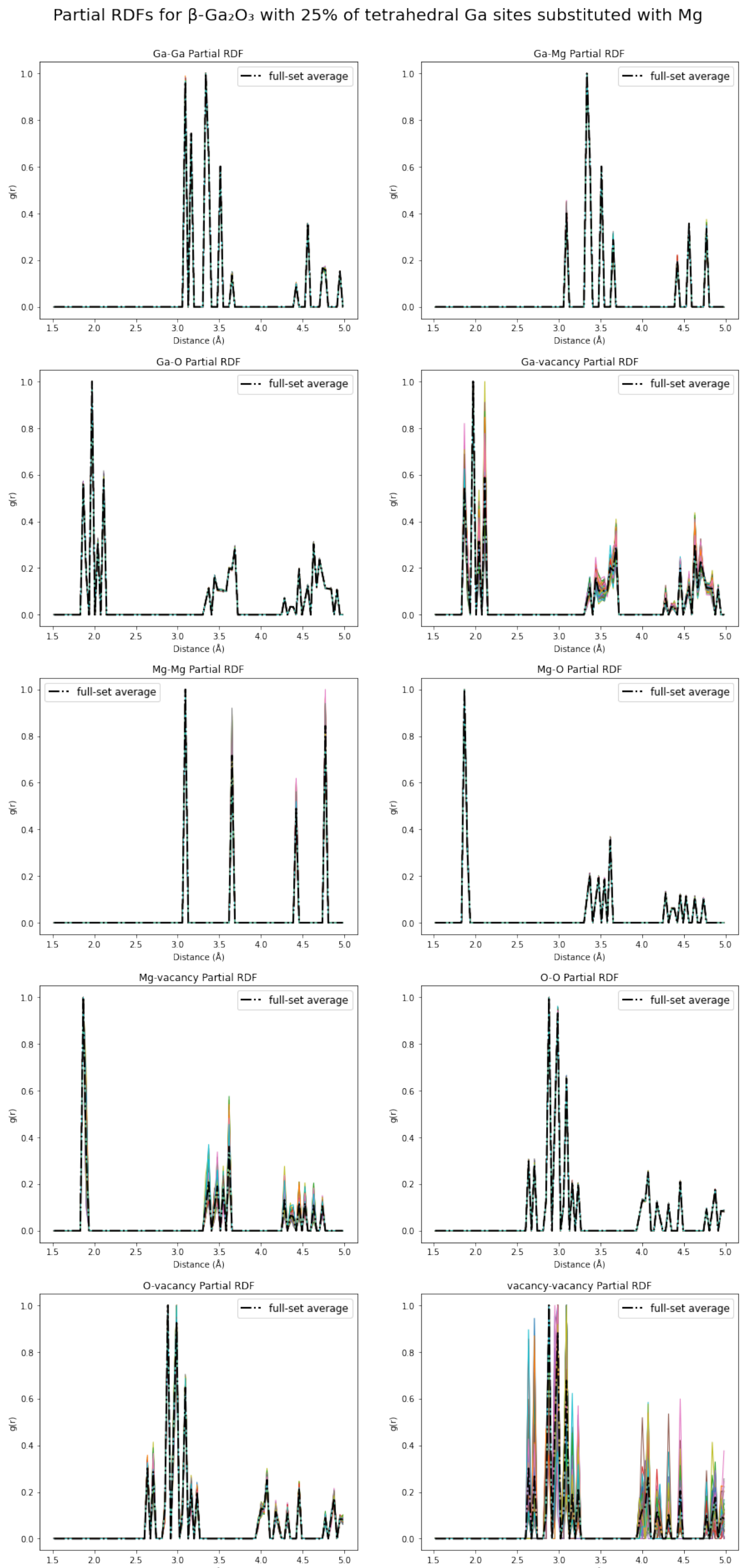

Figure S17: Comparison of average normalized partial radial distribution functions for 100 randomly selected subsets of 10 structures (shown in solid colored lines) with the full-set average from 1000 symmetrically inequivalent structures (dot-dash black line) for β-Ga_2O_3 with 25% of tetrahedral Ga sites substituted by Mg. The RDFs include all relevant atomic pair combinations (Ga–Ga, Ga–Mg, Ga–O, Ga–vacancy, Mg–Mg, Mg–O, Mg–vacancy, O–O, O–vacancy, and vacancy–vacancy). Note: Only distances (x-axis) greater than 1.5 Å are shown for given plots, RDF probabilities are zero for shorter distances.

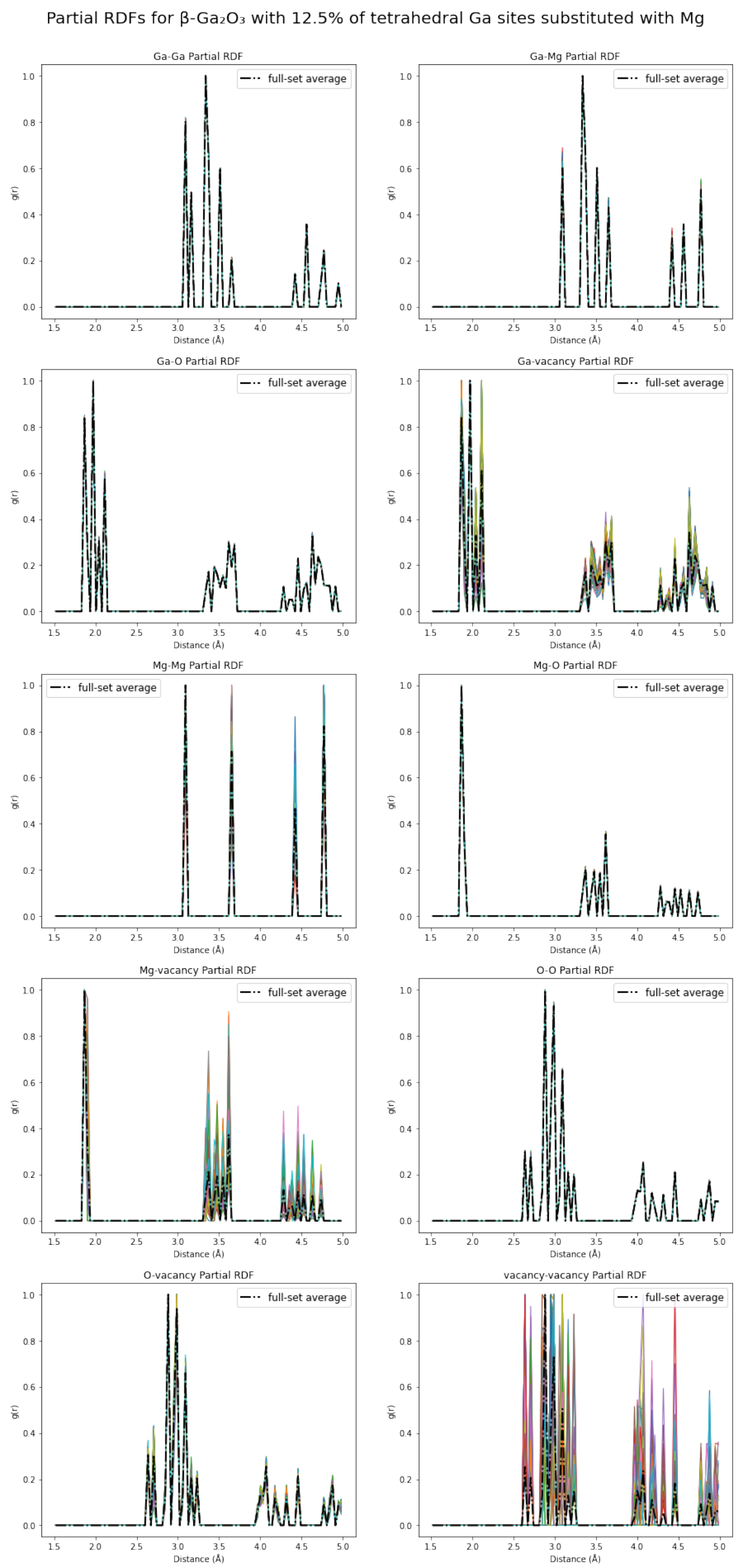

Figure S18: Comparison of average normalized partial radial distribution functions for 100 randomly selected subsets of 10 structures (shown in solid colored lines) with the full-set average from 1000 symmetrically inequivalent structures (dot-dash black line) for β-Ga_2O_3 with 12.5% of tetrahedral Ga sites substituted by Mg. The RDFs include all relevant atomic pair combinations (Ga–Ga, Ga–Mg, Ga–O, Ga–vacancy, Mg–Mg, Mg–O, Mg–vacancy, O–O, O–vacancy, and vacancy–vacancy). Note: Only distances (x-axis) greater than 1.5 Å are shown for given plots, RDF probabilities are zero for shorter distances.

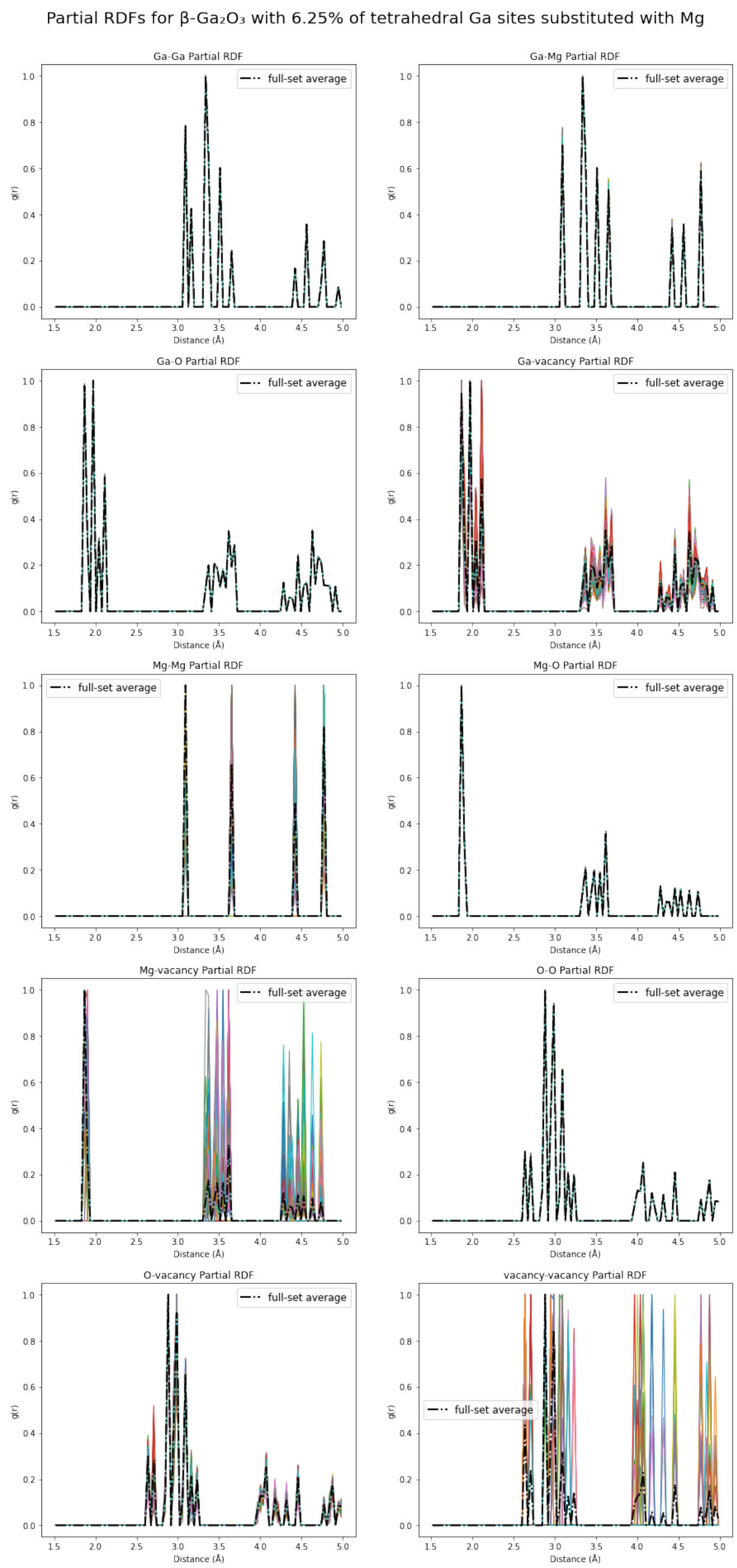

Figure S19: Comparison of average normalized partial radial distribution functions for 100 randomly selected subsets of 10 structures (shown in solid colored lines) with the full-set average from 1000 symmetrically inequivalent structures (dot-dash black line) for β-Ga_2O_3 with 6.25% of tetrahedral Ga sites substituted by Mg. The RDFs include all relevant atomic pair combinations (Ga–Ga, Ga–Mg, Ga–O, Ga–vacancy, Mg–Mg, Mg–O, Mg–vacancy, O–O, O–vacancy, and vacancy–vacancy). Note: Only distances (x-axis) greater than 1.5 Å are shown for given plots, RDF probabilities are zero for shorter distances.

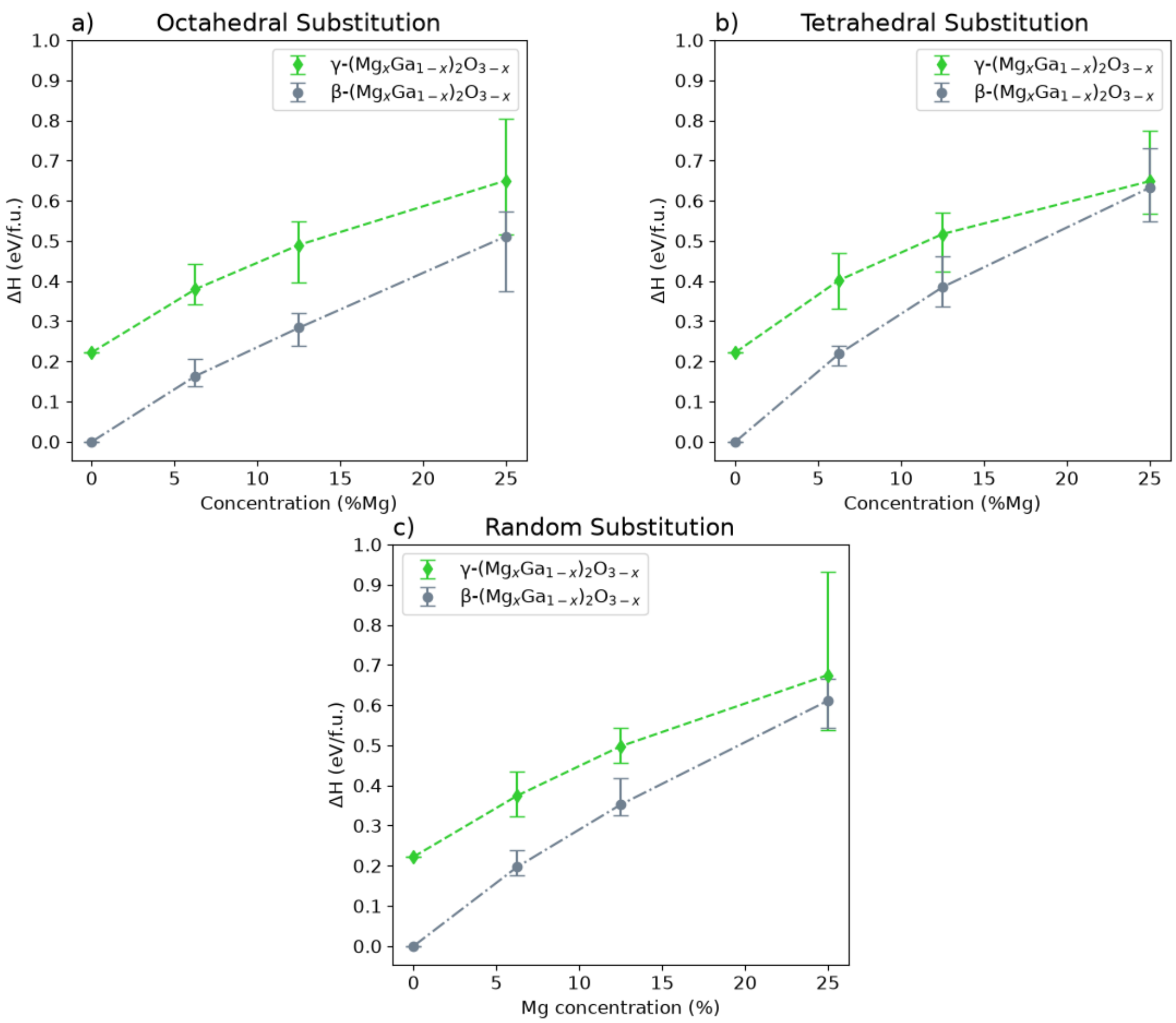

Figure S20: Comparison of the enthalpy of formation difference (ΔH) between Mg-substituted γ-Ga_2O_3 and β-Ga_2O_3 as a function of Mg concentration under three substitution scenarios: (a) octahedral-only, (b) tetrahedral-only, and (c) random distribution.

References

(1) Radial distribution function - Knowledge and References. `https://taylorandfrancis.com/knowledge/Engineering_and_technology/Engineering_support_and_special_topics/Radial_distribution_function/`.

(2) Kullback, S.; Leibler, R. A. On Information and Sufficiency. *The Annals of Mathematical Statistics* **1951**, *22*, 79–86, Publisher: Institute of Mathematical Statistics.
